# Neuronal A_2A_ receptor exacerbates synapse loss and memory deficits in APP/PS1 mice

**DOI:** 10.1093/brain/awae113

**Published:** 2024-07-05

**Authors:** Victoria Gomez-Murcia, Agathe Launay, Kévin Carvalho, Anaëlle Burgard, Céline Meriaux, Raphaëlle Caillierez, Sabiha Eddarkaoui, Devrim Kilinc, Dolores Siedlecki-Wullich, Mélanie Besegher, Séverine Bégard, Bryan Thiroux, Matthieu Jung, Ouada Nebie, Maxence Wisztorski, Nicole Déglon, Claire Montmasson, Alexis-Pierre Bemelmans, Malika Hamdane, Thibaud Lebouvier, Didier Vieau, Isabelle Fournier, Luc Buee, Sabine Lévi, Luisa V Lopes, Anne-Laurence Boutillier, Emilie Faivre, David Blum

**Affiliations:** UMR-S1172 Lille Neuroscience & Cognition (LilNCog), University of Lille, Inserm, CHU Lille, F-59000 Lille, France; Alzheimer & Tauopathies Team, LabEx DISTALZ, University of Lille, F-59000 Lille, France; UMR-S1172 Lille Neuroscience & Cognition (LilNCog), University of Lille, Inserm, CHU Lille, F-59000 Lille, France; Alzheimer & Tauopathies Team, LabEx DISTALZ, University of Lille, F-59000 Lille, France; UMR-S1172 Lille Neuroscience & Cognition (LilNCog), University of Lille, Inserm, CHU Lille, F-59000 Lille, France; Alzheimer & Tauopathies Team, LabEx DISTALZ, University of Lille, F-59000 Lille, France; Laboratoire de Neuroscience Cognitives et Adaptatives (LNCA), University of Strasbourg, F-67000 Strasbourg, France; UMR7364–Laboratoire de Neuroscience Cognitives et Adaptatives (LNCA), CNRS, F-67000 Strasbourg, France; UMR-S1172 Lille Neuroscience & Cognition (LilNCog), University of Lille, Inserm, CHU Lille, F-59000 Lille, France; Alzheimer & Tauopathies Team, LabEx DISTALZ, University of Lille, F-59000 Lille, France; UMR-S1172 Lille Neuroscience & Cognition (LilNCog), University of Lille, Inserm, CHU Lille, F-59000 Lille, France; Alzheimer & Tauopathies Team, LabEx DISTALZ, University of Lille, F-59000 Lille, France; UMR-S1172 Lille Neuroscience & Cognition (LilNCog), University of Lille, Inserm, CHU Lille, F-59000 Lille, France; Alzheimer & Tauopathies Team, LabEx DISTALZ, University of Lille, F-59000 Lille, France; Inserm U1167, LabEx DISTALZ, Université de Lille, Institut Pasteur de Lille, CHU Lille, F-59000 Lille, France; Inserm U1167, LabEx DISTALZ, Université de Lille, Institut Pasteur de Lille, CHU Lille, F-59000 Lille, France; Plateformes Lilloises en Biologie et Santé (PLBS)–UAR 2014–US 41, CNRS, Inserm, Université de Lille, Institut Pasteur de Lille, CHU Lille, F-59000 Lille, France; UMR-S1172 Lille Neuroscience & Cognition (LilNCog), University of Lille, Inserm, CHU Lille, F-59000 Lille, France; Alzheimer & Tauopathies Team, LabEx DISTALZ, University of Lille, F-59000 Lille, France; UMR-S1172 Lille Neuroscience & Cognition (LilNCog), University of Lille, Inserm, CHU Lille, F-59000 Lille, France; Alzheimer & Tauopathies Team, LabEx DISTALZ, University of Lille, F-59000 Lille, France; Institut de Génétique et de Biologie Moléculaire et Cellulaire (IGBMC), University of Strasbourg, CNRS UMR7104, Inserm U1258—GenomEast Platform, F-67400 Illkirch, France; UMR-S1172 Lille Neuroscience & Cognition (LilNCog), University of Lille, Inserm, CHU Lille, F-59000 Lille, France; Alzheimer & Tauopathies Team, LabEx DISTALZ, University of Lille, F-59000 Lille, France; Inserm U1192, Protéomique Réponse Inflammatoire Spectrométrie de Masse (PRISM), Université de Lille, Lille F-59000, France; Laboratory of Cellular and Molecular Neurotherapies (LCMN), Lausanne University Hospital (CHUV) and University of Lausanne (UNIL), Neuroscience Research Center (CRN), 1011 Lausanne, Switzerland; Institut du Fer à Moulin, Inserm UMR-S 1270, Sorbonne Université, F-75005 Paris, France; Laboratoire des Maladies Neurodégénératives: mécanismes, thérapies, imagerie, Université Paris-Saclay, CEA, CNRS, F-92265 Fontenay-aux-Roses, France; UMR-S1172 Lille Neuroscience & Cognition (LilNCog), University of Lille, Inserm, CHU Lille, F-59000 Lille, France; Alzheimer & Tauopathies Team, LabEx DISTALZ, University of Lille, F-59000 Lille, France; UMR-S1172 Lille Neuroscience & Cognition (LilNCog), University of Lille, Inserm, CHU Lille, F-59000 Lille, France; Alzheimer & Tauopathies Team, LabEx DISTALZ, University of Lille, F-59000 Lille, France; Memory Clinic, CHU Lille, F-59000 Lille, France; UMR-S1172 Lille Neuroscience & Cognition (LilNCog), University of Lille, Inserm, CHU Lille, F-59000 Lille, France; Alzheimer & Tauopathies Team, LabEx DISTALZ, University of Lille, F-59000 Lille, France; Inserm U1192, Protéomique Réponse Inflammatoire Spectrométrie de Masse (PRISM), Université de Lille, Lille F-59000, France; UMR-S1172 Lille Neuroscience & Cognition (LilNCog), University of Lille, Inserm, CHU Lille, F-59000 Lille, France; Alzheimer & Tauopathies Team, LabEx DISTALZ, University of Lille, F-59000 Lille, France; Institut du Fer à Moulin, Inserm UMR-S 1270, Sorbonne Université, F-75005 Paris, France; Instituto de Medicina Molecular João Lobo Antunes, Faculdade de Medicina de Lisboa, Universidade de Lisboa, 1649-028 Lisboa, Portugal; Laboratoire de Neuroscience Cognitives et Adaptatives (LNCA), University of Strasbourg, F-67000 Strasbourg, France; UMR7364–Laboratoire de Neuroscience Cognitives et Adaptatives (LNCA), CNRS, F-67000 Strasbourg, France; UMR-S1172 Lille Neuroscience & Cognition (LilNCog), University of Lille, Inserm, CHU Lille, F-59000 Lille, France; Alzheimer & Tauopathies Team, LabEx DISTALZ, University of Lille, F-59000 Lille, France; UMR-S1172 Lille Neuroscience & Cognition (LilNCog), University of Lille, Inserm, CHU Lille, F-59000 Lille, France; Alzheimer & Tauopathies Team, LabEx DISTALZ, University of Lille, F-59000 Lille, France

**Keywords:** adenosine, A_2A_ receptor, Alzheimer’s disease, Synapse loss

## Abstract

Early pathological upregulation of adenosine A_2A_ receptors (A_2A_Rs), one of the caffeine targets, by neurons is thought to be involved in the development of synaptic and memory deficits in Alzheimer’s disease (AD) but mechanisms remain ill-defined. To tackle this question, we promoted a neuronal upregulation of A_2A_R in the hippocampus of APP/PS1 mice developing AD-like amyloidogenesis.

Our findings revealed that the early upregulation of A_2A_R in the presence of an ongoing amyloid pathology exacerbates memory impairments of APP/PS1 mice. These behavioural changes were not linked to major change in the development of amyloid pathology but rather associated with increased phosphorylated tau at neuritic plaques. Moreover, proteomic and transcriptomic analyses coupled with quantitative immunofluorescence studies indicated that neuronal upregulation of the receptor promoted both neuronal and non-neuronal autonomous alterations, i.e. enhanced neuroinflammatory response but also loss of excitatory synapses and impaired neuronal mitochondrial function, presumably accounting for the detrimental effect on memory.

Overall, our results provide compelling evidence that neuronal A_2A_R dysfunction, as seen in the brain of patients, contributes to amyloid-related pathogenesis and underscores the potential of A_2A_R as a relevant therapeutic target for mitigating cognitive impairments in this neurodegenerative disorder.

## Teaser

Gomez-Murcia *et al.* show in a preclinical model of Alzheimer’s disease that early neuronal upregulation of the adenosine A_2A_ receptor, as seen in patients, contributes to the development of memory and synapse loss. The findings underscore the therapeutic potential of A_2A_ receptor blockers, including caffeine, in Alzheimer’s disease.

## Introduction

Alzheimer’s disease (AD) is characterized by a progressive cognitive decline linked to both the extracellular deposition of aggregated amyloid-β (Aβ) peptides into plaques and the intraneuronal aggregation of hyperphosphorylated tau (p-tau) proteins.^1^ AD risk depends on various genetic and environmental factors.^2,3^ Among protective factors, several epidemiological studies have reported an inverse relationship between caffeine intake and both age-related cognitive impairments and the risk of developing AD later in life (for reviews, see Flaten *et al*.,^4^ Cunha^5^ and Yelanchezian *et al*.^6^). In accordance, we and others have shown that caffeine is protective against memory impairments and pathology progression in transgenic mouse models of AD.^7-10^ Based on these studies, we have set up an ongoing placebo-controlled phase III clinical trial (NCT04570085) to evaluate the effect of caffeine on cognitive decline in AD patients at early to moderate stages.

The beneficial effects of caffeine have been ascribed to its ability to block adenosine A_2A_ receptors (A_2A_Rs), a G protein-coupled receptor whose endogenous ligand is adenosine.^5,11^ Indeed, compelling evidence demonstrates that the pharmacological or genetic blockade of A_2A_R mitigates synaptic and memory deficits in various experimental models mimicking the amyloid and/or tau sides of AD.^12-21^ Interestingly, several studies have revealed an exacerbated level, density and activity of A_2A_R at the glutamatergic nerve terminals from the hippocampus of aged animals.^22-25^ Consistently, we demonstrated the neuronal upsurge of A_2A_R in the hippocampus of aged individuals, which was further enhanced in patients with AD.^21^ A_2A_R mRNA upregulation in the brain of AD patients has also been correlated to the clinico-pathological development of the disease,^26^ suggesting it may seemingly be an early event in the AD course.

The benefits of caffeine in AD would, at least in part, rely on its ability to normalize A_2A_R dysfunction in the diseased brain. However, the impact of this neuronal A_2A_R upsurge in the pathophysiological development of AD remains ill-defined. We previously provided data suggesting that upregulation of A_2A_R in neurons favours the development of neuronal tau pathology and drives tau-mediated synaptic loss, through a neuro-microglial miscommunication, involving complement C1q and microglial pruning.^27^ In the present study, we demonstrate that such early neuronal A_2A_R upregulation is also engaged in amyloid synaptotoxicity and related memory deficits in a mouse model of amyloidogenesis but in a microglial phagocytosis-independent manner. These data reinforce the idea that A_2A_R dysfunction plays a role in AD pathogenesis, involving both amyloid and tau-related synaptic and memory deficits by two independent mechanisms, and, hence, may constitute a relevant therapeutic target against AD-related early synaptic loss.

## Materials and methods

### Animals

**Figure 1:**
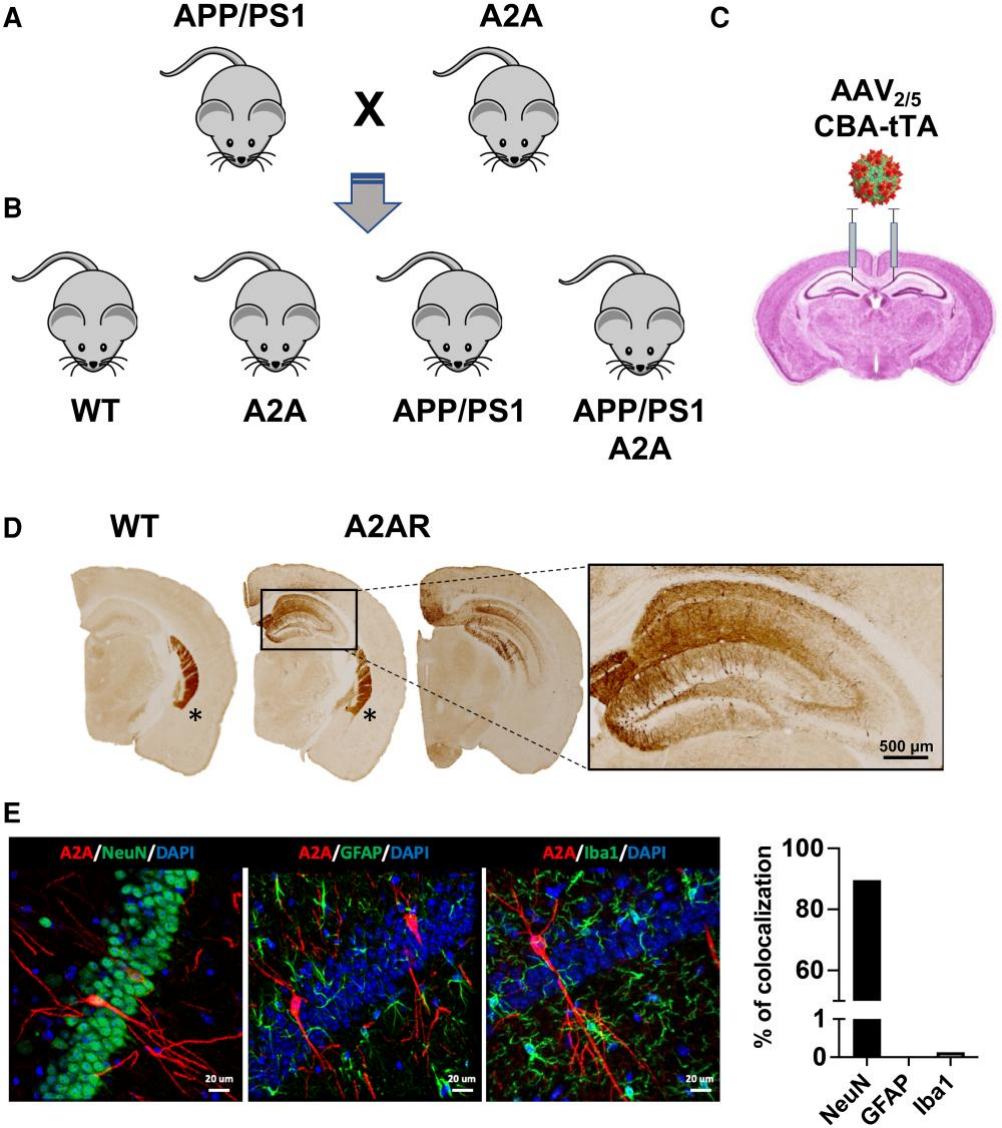
**AAV-based conditional model of neuronal A_2A_ receptor overexpression in the APP/PS1 mouse model of Alzheimer’s disease.** (**A**) APP/PS1 mice were crossed with TRE-A2A (A2A), generating four genotypes, i.e. (**B**) wild-type (WT), TRE-A2A (A2A), APP/PS1 and APP/PS1 TRE-A2A (APP/PS1 A2A). (**C**) The overexpression of A_2A_R in neurons was obtained following a bilateral hippocampal injection of an AAV_2/5_-CBA-tTA-WRPE-bGH in all mice to allow the neuronal expression of the transactivator tTA in all animals. The tTA protein binds to the Tet-responsive element (TRE) allowing the expression of the murine A_2A_R in only TRE-A2A (A2A) and APP/PS1 TRE-A2A (APP/PS1 A2A), WT and APP/PS1 being control groups expressing the transactivator in absence of A_2A_R overexpression. (**D**) Representative pictures of A_2A_R immunohistochemistry where endogenous A_2A_R is found normally highly expressed in the striatum (asterisks) of all mice but overexpressed in the hippocampus, specifically in neuronal cells, of TRE-A2A (A2A) mice injected with the AAV-CBA-tTA viral vector. Scale bar = 500 μm. (**E**) Representative immunofluorescence images showing the co-localization of A_2A_R (red) with NeuN as neuronal marker (green) but absence of co-localization with GFAP astrocytes (green) or Iba1 microglia (green; scale bar = 20 μm). The percentage of cells showing A_2A_R co-localization with these three markers was quantified, showing the selectivity of the neuronal A_2A_R overexpression in the model used.

All animals were maintained in standard cages under conventional laboratory conditions (12 h/12 h light/dark cycle, 22°C), with *ad libitum* access to food and water. Mice were maintained at five to six per cage. The animals were used in compliance with European standards for the care and use of laboratory animals and experimental protocols approved by the local Animal Ethical Committee (Agreement #12787-2015101320441671, CEEA75, Lille, France). Heterozygous APPswe/PS1dE9 mice (herein referred to as APP/PS1, C57Bl6/J background^28^) were crossed with an in-house developed TRE-A2A transgenic strain (in which the mouse A_2A_R cDNA is under the control of a Tet-responsive element^27^ (Fig. 1A). Four genotypic heterozygous groups were therefore obtained: wild-type (WT), TRE-A2A (or A2A), APP/PS1 and APP/PS1 TRE-A2A (or APP/PS1 A2A) (Fig. 1B). Sex dimorphism in the onset time and rate of amyloid pathology, as well as spatial learning and memory impairment, have been described in the APP/PS1 strain, with females more vulnerable than males.^29^ We only used females in the present experiments. Indeed, as A_2A_R expression is known to increase with ageing, we wanted to study the impact of A_2A_R overexpression at a stage when the animals exhibit mild hippocampal amyloid pathology with no memory impairment before any potential increase of endogenous A_2A_R levels, which we ascertained by evaluating A_2A_R mRNA levels by quantitative PCR (qPCR) (Supplementary Fig. 1).

### Viral vectors and stereotaxic injections

To achieve upregulation of neuronal A_2A_R in TRE-A2A mice, a tTA transactivator protein needs to bind the Tet-responsive element (TRE) promoter. Noteworthy, as previously described, an optimized cDNA sequence for A_2A_R, encoding for the same mouse receptor at the protein level, has been used downstream of the TRE promoter,^27^ allowing the respective mRNA level of the transgenic versus endogenous receptor to be distinguished. To this end, a serotype 5 adeno-associated viral (AAV_2/5_) vector, allowing neuronal tropism, encoding the tTA transactivator under the control of the chicken beta-actin (CBA) promoter (AVV_2/5_-CBA-tTA-WPRE-bGH; Fig. 1C), was bilaterally injected into the CA1 region of the dorsal hippocampus of all mice. The tTA protein is expressed in the hippocampus, then binds to the TRE, driving the expression of murine A_2A_R in the TRE-A2A and APP/PS1 TRE-A2A groups only. Control animals, referred as WT and APP/PS1, were injected with the same viral vector but did not express the A_2A_R. All mice were injected at 3 months of age.

For the surgical procedure, mice were deeply anaesthetized with a mixture of ketamine (150 mg/kg) and xylazine (10 mg/kg). Lidocaine (5 mg/kg) was injected subcutaneously under the scalp 10 min before the beginning of surgery. Mice received bilateral stereotaxic injections of viral vectors at a final concentration of 5 × 10^7^ vg/μl in the hippocampus, administered using a 10 μl Hamilton syringe via a 33 gauge blunt needle. The stereotaxic coordinates used were as follows: anteroposterior (AP) −2.5 mm; lateral (L) ±1.0 mm; ventral (V) −1.8 mm from bregma. Mice received a total volume of 2 μl per injection site at a rate of 0.25 μl/min. At the end of the injection, the needle was left in place for 5 min before being removed slowly. The skin was sutured, and mice were allowed to recover.

### Behavioural analysis

Behavioural experiments were conducted at an early stage, between 5 and 6 months of age, before memory impairments were present in the APP/PS1 mouse model. Mice were randomly assigned by experimenters blinded to the genotype, and experiments were performed as per procedures given in the Supplementary material.

### Sacrifice and brain tissue preparation

Mice were euthanized with pentobarbital sodium (50 mg/kg, intraperitoneally) at 6 months of age, then transcardially perfused with 4°C NaCl (0.9%). Brains were removed and divided. For the immunohistochemical studies, one hemisphere was post-fixed for 24 h in 4% paraformaldehyde and cryoprotected in 30% sucrose before being frozen at −40°C in isopentane (methyl-butane) and stored at −80°C. Coronal brain sections (35 µm) were obtained using a Leica cryostat. Free-floating sections were chosen according to the stereological rules, with the first section taken at random and then every 12 sections afterwards, and were stored in PBS-azide (0.2%) at 4°C. The hippocampus of the other hemisphere was dissected out at 4°C and stored at −80°C for biochemical and mRNA analyses. Immunostaining procedures and related analyses are provided in the Supplementary material.

### Biochemical analyses

For all biochemical experiments, mouse tissue was homogenized in 200 µl Tris buffer (pH 7.4) containing 10% sucrose and protease inhibitors (Complete; Roche Diagnostics) and sonicated. Homogenates were kept at −80°C until use. The western blot analysis and ELISA, along with the protein preparation for proteomic analysis, are described in the Supplementary material.

### Nano liquid chromatography-tandem mass spectrometry analysis

The peptides were separated by online reversed-phase chromatography using an EASY-nLC 1000 ultra-high performance liquid chromatography (UPLC) system (Thermo Fisher Scientific) equipped with a 75 µm × 2 cm Acclaim PepMap 100 pre-column with nanoViper fittings (C18, 3 μm, 100 Å, Thermo Fisher Scientific) and a 75 μm × 50 cm Acclaim PepMap rapid separation liquid chromatography (RSLC) analytical column (C18, 100 Å, 2 μm, Thermo Fisher Scientific). Separation was achieved using an increasing amount of acetonitrile (5%–30% over 120 min) at a flow rate of 300 nl/min. Data were acquired on a Thermo Scientific Q-Exactive mass spectrometer set to acquire the top 10 tandem mass spectra in data-dependent mode. The survey scans were carried out at a resolving power of 70 000 full-width at half-maximum (FWHM, m/z 400) in positive mode and using an AGC target value of 3 × 10.^6^ The default charge state was set at 2, unassigned and +1 charge states were rejected and dynamic exclusion was enabled for 20 s. The scan range was set to 300–1600 m/z, one microscan was acquired at 17 500 FWHM, with an isolation window of 4.0 m/z, and a higher energy collision dissociation (HCD) normalized collision energy (NCE) of 30 was used.

### Protein identification and analysis

All MS data were processed with MaxQuant (version 1.6.5.0) using the Andromeda search engine. The proteins were identified by searching MS and tandem MS (MS/MS) data against the reviewed proteome for *Mus musculus* in the UniProt database (Released: March 2019; 17 005 entries). Trypsin specificity was used for the digestion mode. N-terminal acetylation and methionine oxidation were selected as variable- and carbamidomethylation of cysteines as fixed-modifications. Up to two missed cleavages were allowed. An initial mass accuracy of 6 ppm was selected for MS spectra. The MS/MS tolerance was set to 20 ppm. The false discovery rate at the peptide spectrum matches and protein level was estimated using a decoy version of the previously defined databases (reverse construction) and set to 1%. Relative label-free quantification (LFQ) of the proteins was conducted in MaxQuant using the MaxLFQ algorithm with default parameters. The file containing the information about the identification of proteins was used for the analysis by Perseus software (http://www.perseus-framework.org, version 1.6.5.0). Hits from the reverse database, proteins with only modified peptides and potential contaminants were removed. The LFQ intensities were transformed by logarithm base 2. Unsupervised multivariate analysis was performed using principal component analysis (PCA). Statistical analysis of the difference between experimental groups was performed using Student’s *t*-test, with a two-tailed test and a *P*-value <0.05 considered statistically significant. Hierarchical clustering was performed only with the proteins presenting a statistically significant *P*-value and a log2 fold-change value <−0.32 and >0.32, using the Euclidean parameter for distance calculation, the average option for linkage in row and column trees and a maximum of 300 clusters. Functional protein association networks were obtained using STRING (version 11.0, http://string-db.org).

### mRNA extraction and real-time quantitative reverse transcription-PCR analysis

Total RNA was extracted from hippocampi and purified using the RNeasy Lipid Tissue Mini Kit (Qiagen). qPCR analyses are described in the Supplementary material.

### RNA-sequencing

RNA-sequencing (RNS-seq) libraries were generated from 500 ng of total RNA using a TruSeq Stranded mRNA LT Sample Preparation Kit (Illumina), according to the manufacturer’s instructions. Briefly, following purification with poly-T oligo attached magnetic beads, the mRNA was fragmented using divalent cations at 94°C for 2 min. The cleaved RNA fragments were copied into first strand cDNA using reverse transcriptase and random primers. Strand specificity was achieved by replacing dTTP with dUTP during second strand cDNA synthesis using DNA polymerase I and RNase H. Following addition of a single ‘A’ base and subsequent ligation of the adapter on double stranded cDNA fragments, the products were purified and enriched with PCR [30 s at 98°C; (10 s at 98°C, 30 s at 60°C, 30 s at 72°C) × 12 cycles; 5 min at 72°C] to create the cDNA library. Surplus PCR primers were further removed by purification using AMPure XP beads (Beckman-Coulter) and the final cDNA libraries were checked for quality and quantified using capillary electrophoresis. Sequencing was performed on an Illumina HiSeq 4000 in a 1 × 50 bp single read format following Illumina’s instructions. Reads were then preprocessed using cutadapt 1.10^30^ to remove adaptors and low-quality sequences and reads shorter than 40 bp were removed for further analysis. Remaining reads were mapped to *M. musculus* rRNA sequences using bowtie 2.2.8^31^ and reads mapped to those sequences were removed for further analysis. Remaining reads were aligned to the mm10 assembly of *M. musculus* with STAR 2.5.3a.^32^ Gene quantification was performed with htseq-count 0.6.1p1,^33^ using ‘union’ mode and Ensembl 94 annotations. Differential gene expression analysis was performed using DESeq2 1.16.1^34^ Bioconductor R package on previously obtained counts (with default options). *P*-values were adjusted for multiple testing using the Benjamini and Hochberg method.^35^ Sequencing data that support the findings of this study have been deposited in the NCBI’s Gene Expression Omnibus (GEO) database (GSE248245). Four to five biological replicates were used per group and the PCA is provided in Supplementary Fig. 2. To identify different gene co-expression modules in our RNA-seq data, we used the Co-Expression Modules identification Tool (CEMiTool)^36^ with normalized expression values from DESeq2 analysis (variance filter *P*-value: 0.2; variance stabilizing transformation: TRUE; value of beta: 8), resulting in 16 correlated modules (Fig. 5A). *Z*-score expression for violin plot representation of RNA-seq data from the different modules was created with R software.

### Statistical analysis

Image acquisition and quantification as well as behavioural evaluations were performed by investigators blind to the experimental conditions. Results are expressed as means ± standard error of the mean. Differences between mean values were determined using the one-sample *t*-test, two-tailed unpaired Student’s *t*-test, two-way ANOVA or one-way ANOVA, followed by a *post hoc* Tukey’s multiple comparisons test using Graphpad Prism software. *P*-values <0.05 were considered statistically significant.

## Results

### Neuronal overexpression of A_2A_R worsens spatial memory deficits in APP/PS1 mice

To determine the impact of early A_2A_R neuronal upregulation, as seen in the aged and AD human brain,^21^ on the pathophysiological development of the APP/PS1 amyloid mouse model, we took advantage of a conditional strain that we developed recently.^27^ This model, carrying the mouse A_2A_R transgene under the control of a Tet-responsive element (TRE-A2A strain), was crossed with APP/PS1 mice (Fig. 1A). The resulting WT, TRE-A2A (or A2A), APP/PS1 and APP/PS1 TRE-A2A (or APP/PS1 A2A) (Fig. 1B) were bilaterally injected in the hippocampus with an AAV_2/5_ viral vector allowing the expression of the tTA transactivator protein in neurons (Fig. 1C).^37,38^ All animals were injected with the viral vector at the age of 3 months and subsequent evaluations were performed at 6 months of age, an early time point of pathological development in this mouse model, when there are no behavioural alterations.^39,40^ Figure 1D describes the topology of upregulated A_2A_R expression in the dorsal hippocampus of TRE-A2A versus WT animals. Double stainings against A_2A_R and either NeuN, GFAP or Iba1 revealed that A_2A_R overexpression occurred most exclusively in neurons (Fig. 1E). Noteworthy, mRNA of the transgenic receptor was only detected in A2A animals (WT A2A or APP/PS1 A2A), at a similar level and, as expected, remained undetectable (ND) in control animals (WT and APP/PS1; Supplementary Fig. 1, upper panel). The level of the endogenous A_2A_R mRNA remained similar regardless the experimental group, supporting an absence of compensation (Supplementary Fig. 1, lower panel).

**Figure 2:**
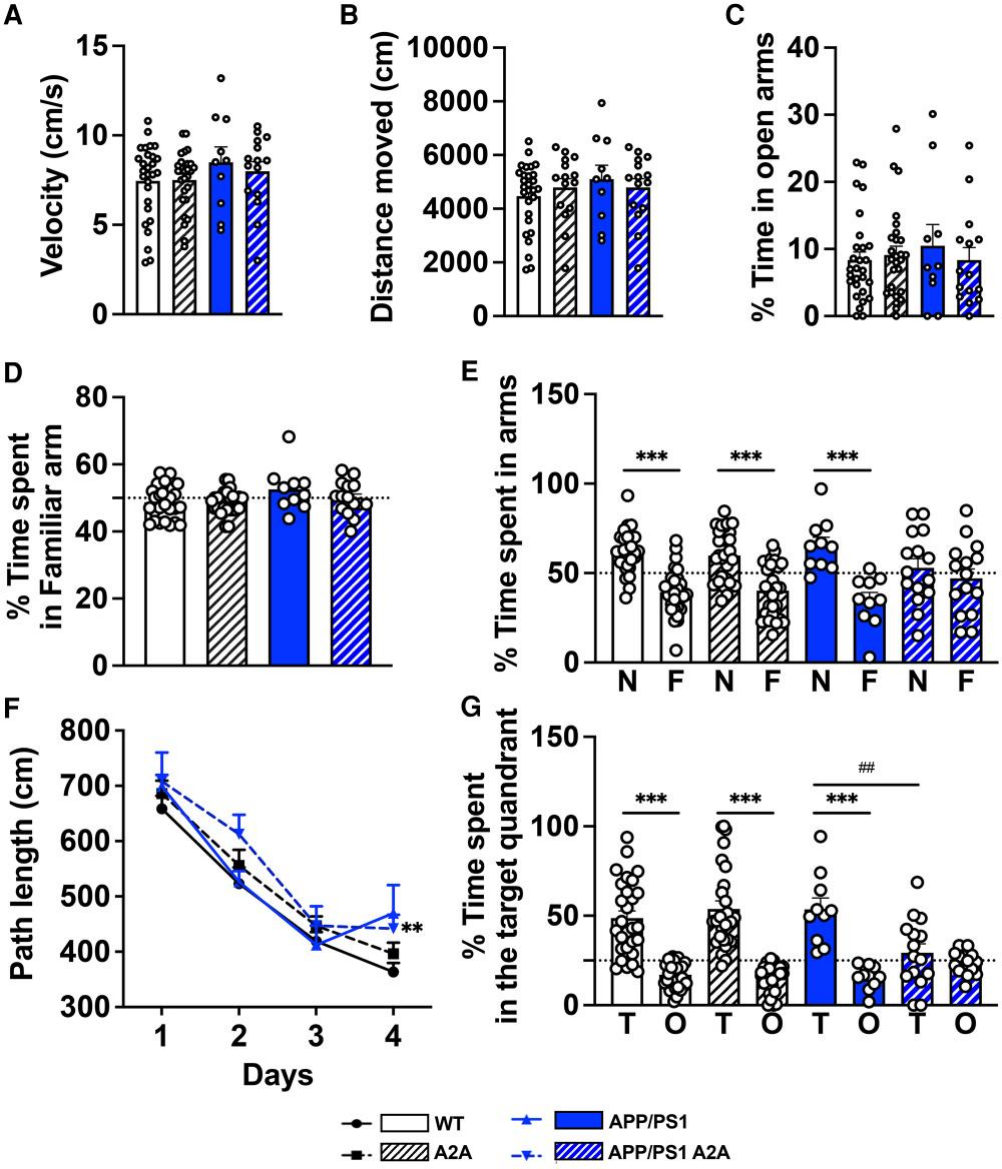
**Early neuronal upregulation of A_2A_ receptors favours memory deficits of APP/PS1 transgenic mice.** Effects of neuronal overexpression of A_2A_R on spontaneous activity (**A** and **B**), anxiety-like behaviour (**C**), spatial learning and memory (**D**–**G**) of APP/PS1 mice. (**A** and **B**) No difference in spontaneous locomotion (velocity, **A**; distance moved, **B**) was found between groups using actimetry. (**C**) In the elevated plus maze, no difference in the percentage of time spent in the open arms was found between groups. (**D** and **E**) Y-maze task. During the learning phase (**D**; dashed line represents 50%), mice spent a similar percentage of time in the familiar arm versus the start arm. In the test phase (**E**), wild-type (WT), A2A and APP/PS1 mice exhibited preference for the new arm (N) compared with the familiar arm (F), while the APP/PS1 A2A group showed spatial memory deficits, attested by the absence of preference for the N over the F arm. ***P < 0.001 versus new arm using one-way ANOVA followed by Tukey’s *post hoc* test. Dashed line represents 50%, i.e. chance. (**F** and **G**) Barnes-maze task. During the acquisition phase, all mice properly learned the location of the target hole as attested by the decreased of path length across trials, with a slight learning deficit in APP/PS1 A2A mice on Day 4. **P < 0.01 versus WT (two-way ANOVA). (**G**) Spatial memory was assessed 24 h after the last training session. WT, A2A and APP/PS1 mice spent a significantly higher amount of time in the target quadrant (T) versus other quadrants (O). ***P < 0.001 versus other quadrants using one-way ANOVA followed by Tukey’s *post hoc* test. In contrast, APP/PS1 A2A mice exhibited no preference for the T versus O quadrants, supporting memory deficit. ^##^*P* < 0.01 versus APP/PS1, one-way ANOVA followed by Tukey’s *post hoc* test. *n* = 10–28 mice per group.

Using actimetry and an elevated plus maze, no difference in either spontaneous activity (velocity and distance moved, *P* > 0.05; Fig. 2A and B) nor anxiety-like behaviour (*P* > 0.05; Fig. 2C) could be observed between the different groups. Then, we evaluated the impact of the A_2A_R hippocampal neuronal overexpression on spatial memory. In a first attempt, we determined the effects on short-term spatial memory using the Y-maze task. During the acquisition phase, all groups explored the maze equally, spending a similar amount of time (50%; dashed line on Fig. 2D) exploring the familiar arm (*P* > 0.05; Fig. 2D). During the test phase, animals from the WT, A2A and APP/PS1 groups exhibited a preference for the novel arm versus the familiar arm [F(7151) = 10.83, *P* < 0.0001; one-way ANOVA followed by Tukey’s *post hoc* test; Fig. 2E), demonstrating efficient memory. Accordingly, the preference of mice for the novel versus the familiar arm was significantly above chance (i.e. 50%; dashed line on Fig. 2E) for WT [61.8 ± 2.3%; *t*(26) = 5.163, *P* < 0.0001; one-sample *t*-test], A2A [59.9 ± 2.8%; *t*(26) = 3.529, *P* = 0.0016] and APP/PS1 [65.4 ± 4.6%; *t*(9) = 3.368, *P* = 0.0083] animals (Fig. 2E). In sharp contrast, APP/PS1 A2A mice did not show preference for the new over the familiar arm (*P* = 0.96; one-way ANOVA followed by Tukey’s *post hoc* test), with a percentage of time spent in the novel arm at the level of chance (52.9 ± 5.1%; *P* = 0.58; one-sample *t*-test; Fig. 2E).

Whenever evaluating long-term spatial memory, using the Barnes maze task, all groups showed a decreased path length across trials [*F*(3302) = 86.10, *P* < 0.001; two-way ANOVA; Fig. 2F] during the learning phase. A2A and APP/PS1 mice demonstrated similar learning compared with the WT animals, whereas APP/PS1 A2A animals exhibited a slight but significant decrease in learning over time (*P* < 0.01; two-way ANOVA; Fig. 2F). Twenty-four hours following the last learning trial, a probe trial was performed to assess spatial memory. WT, A2A and APP/PS1 mice exhibited a significant preference for the target (T) quadrant over the other (O; non targets) quadrants [*F*(7152) = 24.08, *P* < 0.0001; one-way ANOVA followed by Tukey’s *post hoc* test; Fig. 2G] and spent a significantly greater proportion of time in the former than expected by chance [i.e. 25%; dashed line on Fig. 2G; WT: 48.7 ± 3.9%, *t*(27) = 5.935, *P* < 0.0001; A2A: 53.9 ± 4.7%, *t*(26) = 6.460, *P* < 0.0001; APP/PS1: 53.6 ± 6.3%, *t*(9) = 4.519, *P* = 0014; one-sample *t*-test]. In contrast, the APP/PS1 A2A mice exhibited no preference for the target quadrant (*P* = 0.97; one-way ANOVA followed by Tukey’s *post hoc* test) and the percentage of time spent in this quadrant was at the chance level (29.3 ± 4.9%; *P* = 0.39; one sample *t*-test; Fig. 2G). Together, these data suggest that, at an early stage of pathological development, neuronal A_2A_R upregulation significantly potentiates the development of spatial memory impairments in APP/PS1 mice, which are normally observed at later stages.

### Impact of neuronal overexpression of A_2A_R on hippocampal pathology of APP/PS1 mice

**Figure 3:**
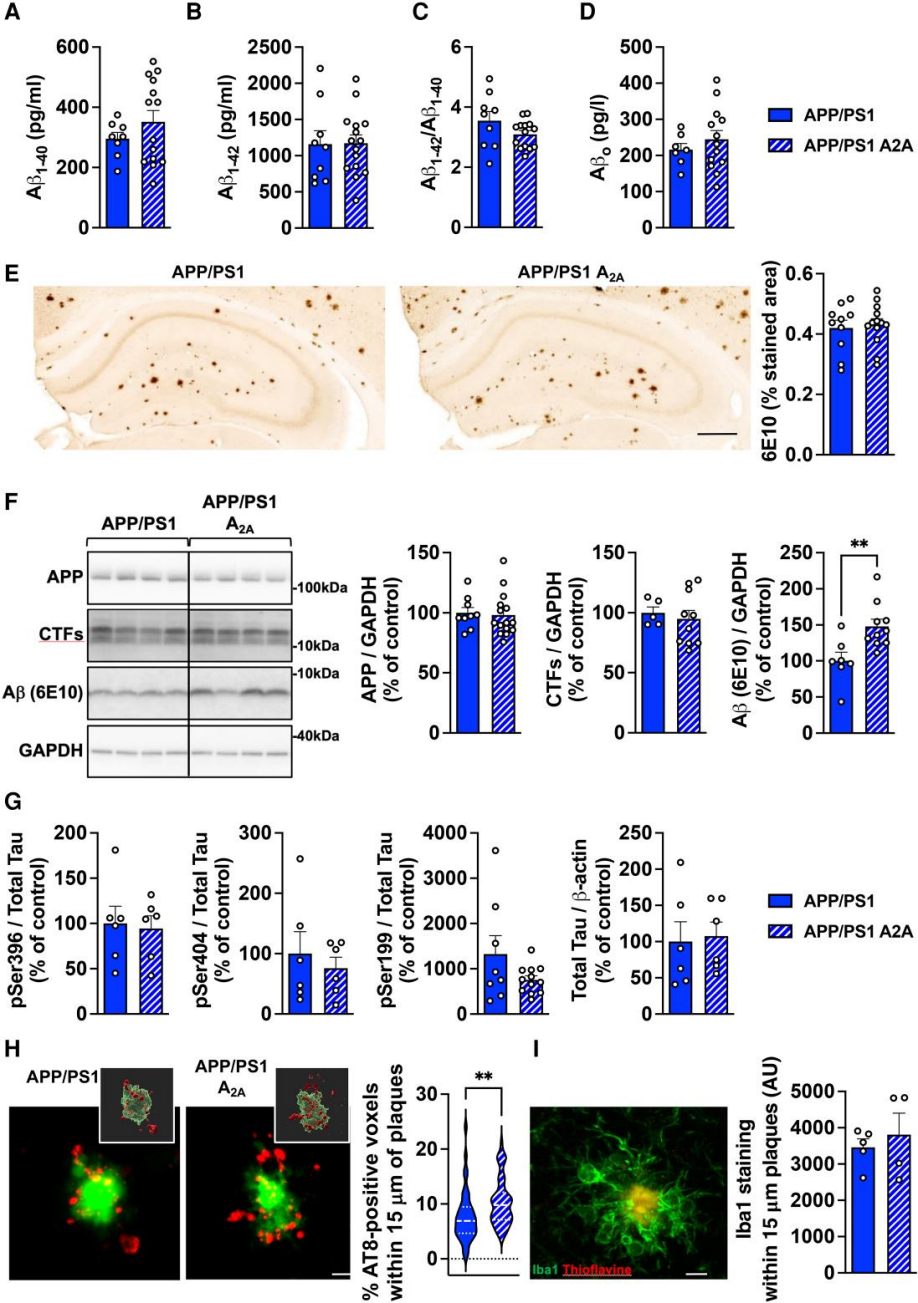
**Neuronal upregulation of A_2A_ receptors in APP/PS1 mice increases hippocampal phosphorylated tau at neuritic plaques with a limited impact on amyloid pathology**. (**A**–**D**) Evaluation of amyloid pathology. Hippocampal levels of amyloid-β (Aβ)_1–40_, Aβ_1–42_, Aβ_1–40_/Aβ_1–42_ ratio and Aβo were assessed by ELISA with no differences observed between groups. *n* = 9–14 mice per group. (**E**) Representative images of 6E10-positive amyloid plaques in the brains of APP/PS1 and APP/PS1 A2A mice (scale bar = 500 µm). Quantification of hippocampal Aβ plaque load showed no difference between mice. *n* = 10–14 mice per group. (**F**) Western blot evaluation of APP, C-terminal fragments (CTFs) and total Aβ (6E10) in the hippocampus of APP/PS1 mice and APP/PS1 A2A mice. An increase of total Aβ levels was found in APP/PS1 A2A versus APP/PS1 mice. **P < 0.01, Student’s *t*-test. Evaluation of tau pathology. *n* = 5–15 mice per group. (**G**) Hippocampal levels of pSer396, pSer404 assessed by western blot as well as pSer199 and total tau assessed by ELISA with no differences observed between groups. *n* = 6–12 mice per group. (**H**) Confocal images and relative 3D surface rendering showing volume reconstruction of hippocampal AT8^+^ tau neuritic plaques (NP, red) around thioflavin^+^ amyloid plaques (green; scale bar = 10 µm). Quantification of the percent of AT8^+^ voxels within 15 μm of plaques showed an increased in APP/PS1 A2A versus APP/PS1 mice. **P < 0.01. Student’s *t*-test. *n* = 45–61 plaques from four to five mice per group. (**I**) Representative immunofluorescence of Iba1^+^ (green) microglial staining around Thioflavine^+^ plaque (red; scale bar = 10 µm) and quantification of Iba1 staining within 15 μm of plaques. *n* = 4–5 mice per group.

As spatial memory impairments in APP/PS1 mice primarily relate to amyloid burden and considering previous studies suggesting that A_2A_R might regulate Aβ production and pathology,^17,41^ we then characterized hippocampal amyloid pathology in APP/PS1 A2A versus APP/PS1 mice. We could not evidence change in the levels of Aβ_1–40_, Aβ_1–42_, Aβ_1–40_/Aβ_1–42_ or Aβ_o_ using ELISA (*P* > 0.05, Student’s *t*-test; Fig. 3A–D). Using 6E10 immunohistochemistry, we analysed the hippocampal Aβ plaque load and found no difference between APP/PS1 and APP/PS1 A2A mice (*P* > 0.05, Student’s *t*-test; Fig. 3E). Western blot analysis showed that A_2A_R neuronal upsurge altered neither human APP expression nor APP C-terminal fragments (CTFs) levels in APP/PS1 mice (*P* > 0.05, Student’s *t*-test; Fig. 3F). However, the level of total Aβ, detected after 6E10-immunoblotting, was found moderately but significantly increased in APP/PS1 A2A versus APP/PS1 mice [+48.0 ± 3.1%; *t*(15) = 3.090, *P* = 0.0075; Student’s *t*-test; Fig. 3F]. Considering the pathophysiological link between amyloid and tau in AD^1^ and our recent demonstration that neuronal A_2A_R upregulation impacts tau phosphorylation in a mouse model of tauopathy,^27^ we also evaluated the levels of murine phospshorylated tau (p-tau) and total tau in the hippocampal parenchyma of APP/PS1 and APP/PS1 A2A mice and found no significant differences (*P* > 0.05, Student’s *t*-test; Fig. 3G). However, we found a significant rise of AT8-positive area surrounding amyloid deposits, i.e. at neuritic plaques, in APP/PS1 A2A versus APP/PS1 mice [+34.2 ± 7.3.1%; *t*(97) = 2.904, *P* = 0.0046; Student’s *t*-test; Fig. 3H] without any change in the levels of Iba1^+^-microglial surrounding amyloid plaques (*P* > 0.05, Student’s *t*-test; Fig. 3I). These changes occurred in the absence of gross changes in hippocampal morphology and layer thickness (not shown). Overall, we found a singular change of endogenous p-tau at neuritic plaques in APP/SP1 A2A animals but a limited impact of neuronal A_2A_R overexpression on the accumulation of amyloid peptides.

### Transcriptomic signature associated with the neuronal overexpression of A_2A_R in APP/PS1 mice

**Figure 4:**
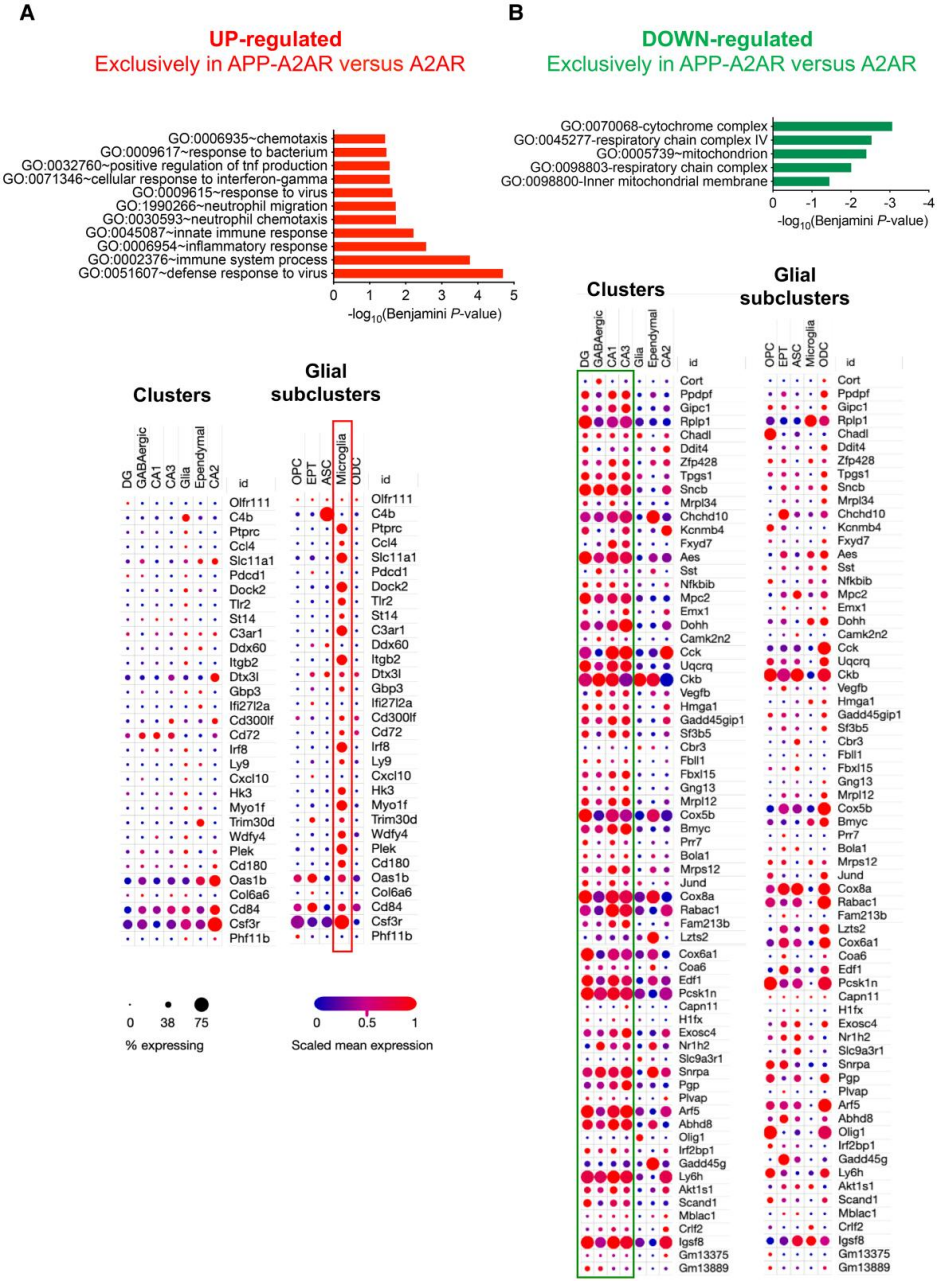
**Transcriptomic signatures associated with the upregulation of A_2A_ receptors in the hippocampus of APP/PS1 mice.** (**A** and **B**) Upregulated and downregulated genes. *Top*: Functional annotation, performed with the Database for Annotation, Visualization and Integrated Discovery (DAVID), of the 35 upregulated genes and 79 downregulated genes specifically found in APP/PS1 A2A versus A2A (Supplementary Fig. 5). Notably, upregulated genes significantly associate with immune-related processes (*top left*) and downregulated genes with mitochondrial functions (*top right*). *Bottom*: Integration of the significantly dysregulated genes from the *top* with single nucleus RNA sequencing performed in the adult mouse hippocampus by the Broad Institute.^42^ Gene lists were analysed first taking into account major hippocampal cell types [clusters: granule cells from the dentate gyrus; pyramidal neurons from CA1, CA2 and CA3; GABAergic neurons; glia-like cells; and ependymal cells and then glial subclusters: oligodendrocyte progenitor cells (OPC); epithelial cells (EPT); astrocytes (ASC); microglial cells; and oligodendrocytes (ODC)]. Association with a specific cellular subtype is shown as a dot plot. The intensity of the colour (blue to red) represents the level of gene expression and the size of each dot represents the percentage of cells expressing the gene for a given annotation selection.

To gain mechanistic insights on how neuronal A_2A_R overexpression might affect memory of APP/PS1 transgenic mice, we performed a hippocampal RNA-seq analysis from the different groups of animals at the age of 6 months. According to the early stage considered, a limited number of differentially expressed genes was found in APP/PS1 compared with WT mice, with only 21 differentially expressed genes (|Log2 fold-change| > 0.32, *P*_adjusted_ < 0.05; Supplementary Fig. 5 and Supplementary Table 3), all upregulated in APP/PS1 mice and most related to neuroinflammatory processes (e.g. *Gfap*, *Trem2*, *Cd68*, *Tyobp*, *Ccl3*, *Clec7a*, *Ccl6*, *Itgax*, *Cst7*; Supplementary Fig. 5). Neuronal overexpression of A_2A_R itself did not lead to transcriptomic changes compared with WT animals (*P* > 0.05; not shown), in agreement with our previous findings.^27^ However, the impact of the APP/PS1 genotype was found to be stronger under the A2A background. Indeed, we found 130 differentially expressed genes (i.e. ∼6-fold more than in APP/PS1 versus WT) between APP/PS1 A2A versus A2A animals (|Log2 fold-change|>0.32, *P*_adjusted_ < 0.05), 51 being upregulated (in red) and 79 downregulated (Supplementary Fig. 5 and Supplementary Table 3). Among these 130 genes, 114 were exclusively altered in APP/PS1 A2A mice. Among the 51 upregulated genes, 16 were common to the 21 upregulated in APP/PS1 versus WT mice, with the fold-change being similar (Supplementary Fig. 5 and Supplementary Table 3). The 35 genes specifically upregulated in the APP/PS1 A2A mice were associated with immune processes (Fig. 4A, top left, and Supplementary Fig. 6A, STRING analysis). To gain insight into the enrichment of these genes with regard to cellular expression, we compared our bulk RNA-seq data with the single-cell hippocampal RNA-seq (scRNA-seq) data provided by the Broad institute.^42^ The data in Fig. 4A (bottom) clearly indicate that most of the exclusive upregulated genes are expressed by microglial cells. Regarding the genes downregulated in APP/PS1 A2A animals, functional biological process annotations indicated an enrichment in genes associated with mitochondrial function, respiratory chain and in particular complex IV (Fig. 4B, top right, and Supplementary Fig. 6B, STRING analysis). Comparison with the scRNA-seq data highlighted that these changes were more likely ascribed to neuronal cells with few genes modulated in the glial subcluster (Fig. 4B, bottom). We also compared the set of 114 genes exclusively changed in APP/PS1 A2A and A2A mice (Supplementary Fig. 4) with hippocampal signatures already published in APP/PS1 mice and AD patients. First, the APP/PS1 A2A signature has been compared to a list of genes differentially expressed in APP/PS1 mice at 18 versus 7 months of age,^43^ i.e. genes that indicate the pathophysiological evolution of this transgenic mouse model. We found 63 common genes sharing similar variation with APP/PS1 A2A mice (Supplementary Fig. 7). Among these 63 genes, 24 were upregulated as in the APP/PS1 A2A mice (Supplementary Fig. 7). Gene ontology (GO) term (Database for Annotation, Visualization and Integrated Discovery, DAVID) and STRING analyses indicated that these genes were associated with immune signatures (Supplementary Fig. 7). The 39 genes commonly downregulated in evolving APP/PS1 and APP/PS1 A2A mice were associated with mitochondrial pathways (Supplementary Fig. 7). Similar observations were made when we compared the 114 exclusive hippocampal genes modulated in APP/PS1 A2A mice to the hippocampal transcriptome of AD patients^44^ (Supplementary Fig. 8).

**Figure 5:**
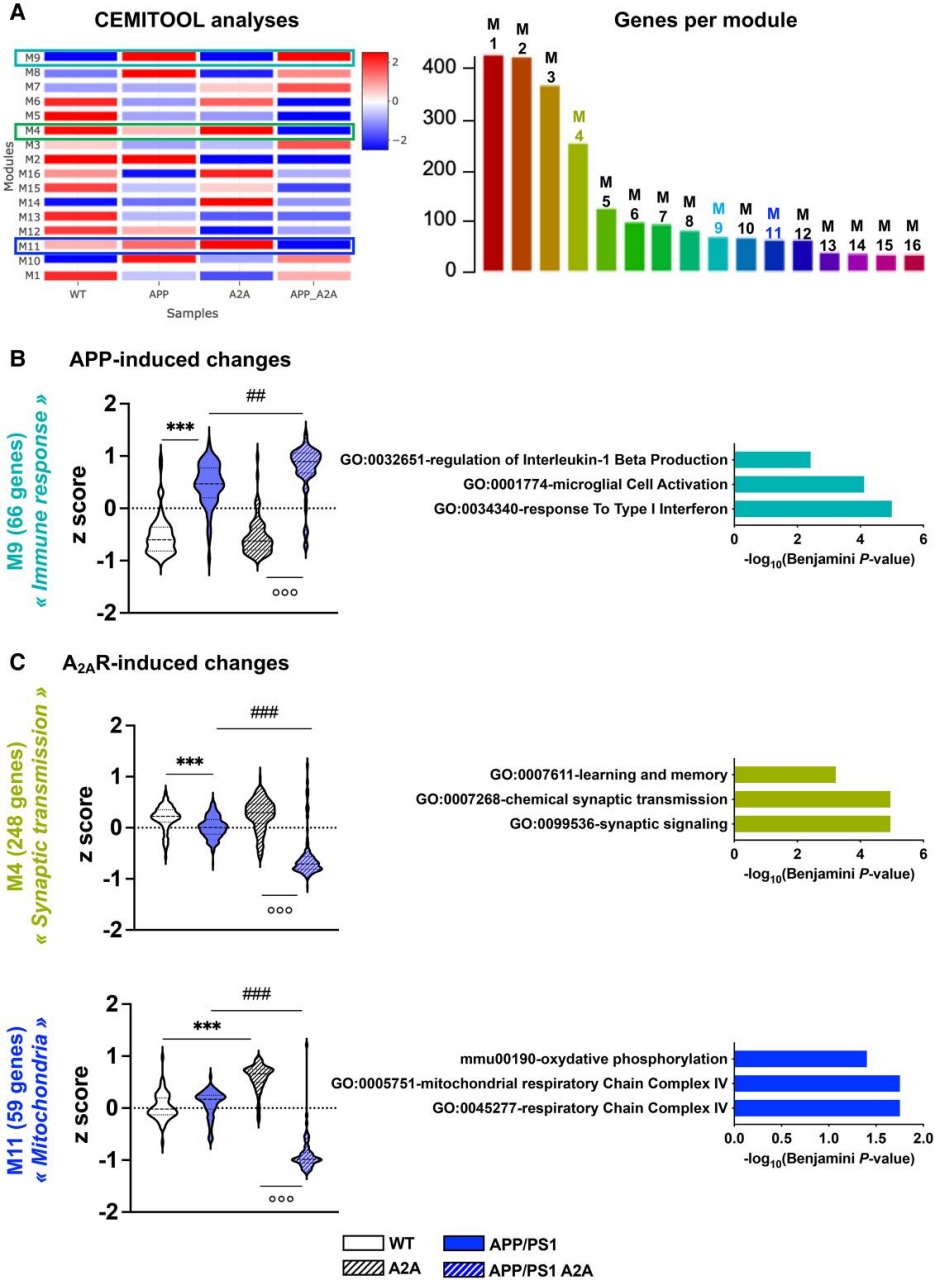
**Identification of co-expression modules in the RNA-sequencing data.** (**A**) Heat map showing modules normalized enrichment score (NES) identified by CEMiTool analysis. These represent co-expressed genes. Red and blue represent, respectively, higher and lower module activity. The three modules analysed in **B** and **C** are highlighted. The number of genes belonging to each identified module is shown on the *right*. (**B**) Violin plots representing *z*-score expression values of genes from the RNA sequencing analyses in the different genotypes (*n* = 4 per group) defined by the M9 module associated with the ‘Immune response’ signature. This module was upregulated in both APP/PS1 and APP/PS1 A2A hippocampi, indicating an APP/PS1-dependent modulation. (**C**) Violin plots representing *z*-score expression values of genes from the RNA sequencing analyses in the different genotypes (*n* = 4 per group) defined by the M4 module associated with the ‘synaptic transmission’ signature and the M11 module associated with the ‘Mitochondria’ signature. In both modules, genes expressed in the APP/PS1 A2AR condition exhibited severe downregulation compared with other genotypes, indicating A2AR-induced changes in the APP/PS1 background. ***^,###,^°°°*P* < 0.001 and **^,##^*P* < 0.01 using the non-parametric Kruskal–Wallis test and original false discovery rate method of Benjamini and Hochberg for the *post hoc* test. Pathway analysis (Enrichr, Biological Process) of dysregulated genes in the associated module is represented by the three most significant terms on the *right*.

**Figure 6:**
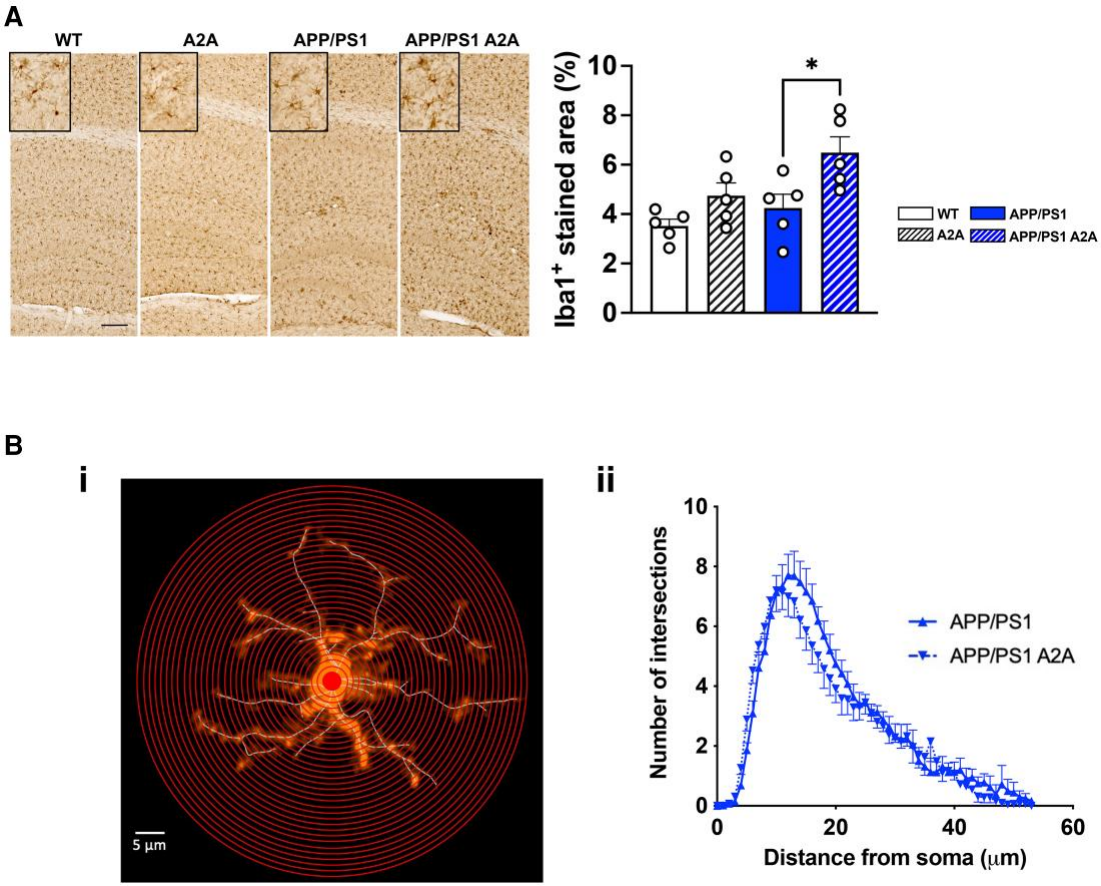
**Analysis of microglial cell in APP/PS1 A2A mice.** (**A**) Representative images of Iba1 immunostaining of the hippocampus of wild-type (WT), A2A, APP/PS1 and APP/PS1 A2A animals (scale bar = 200 µm). Quantification of Iba+ stained area showed a significant increase in APP/PS1 A2A versus APP/PS1 mice. *P < 0.05, one-way ANOVA followed by Tukey’s *post hoc* test. *n* = 5 mice per group. (**B**) Sholl analysis of Iba1-immunolabelled microglia. [**B**(**i**)] Sholl analysis of reconstructed microglia was performed by placing a series of concentric circles spaced at 1 μm intervals and centered on the soma. [**B**(**ii**)] Plot of the number of microglia process intersections as a function of the radial distance from the soma. We found that the complexity of microglial processes was significantly reduced in APP/PS1 A2A versus APP/PS1 mice (*P* = 0.0031, two-way ANOVA).

In addition to these analyses, we performed an unsupervised analysis using CEMiTool (https://cemitool.sysbio.tools/; Fig. 5),^36^ which identified 2202 transcripts within 16 modules that were co-regulated in the hippocampus of APP/PS1 A2A mice (Fig. 5A). We particularly selected three modules (M9, M4 and M11) according to their relevance to AD pathology and A_2A_R dependency. The APP/PS1 genotype induced a significant upregulation of genes from the M9 module ‘immune response’ (linked to microglial, Il-1β and interferon-related pathways; Fig. 5B, right). In agreement with our supervised analysis, this effect was exacerbated by the joint presence of A_2A_R. Such exacerbation was in agreement with the rise of Iba1^+^ immunostaining found in the hippocampal parenchyma of APP/PS1 A2A mice [*F*(3,16) = 5.897, *P* = 0.0066; APP/PS1 versus APP/PS1 A2A, *P* = 0.034; one-way ANOVA followed by Tukey’s *post hoc* test; Fig. 6A] as well as with the reduced ramification complexity of parenchymal microglia (localized at least >50 μm from plaques) found in APP/PS1 A2A versus APP/PS1 mice using Sholl analysis [*F*(1,504) = 8.840, *P* = 0.0031; two-way ANOVA; Fig. 6B]. Noteworthy, we also evaluated the mRNA levels of astrocytic genes and found no impact of A_2A_R neuronal overexpression in APP/PS1 mice (Supplementary Fig. 9). Interestingly, whereas the presence of A2AR or APP/PS1 did not alter or moderately altered gene expression in the M4 (‘Synaptic transmission’, linked to GO term memory and synaptic pathways) and M11 modules (‘Mitochondria’, linked to GO term oxidative phosphorylation and complex IV pathways), the neuronal A_2A_R upregulation in APP/PS1 mice induced a strong downregulation of these genes (Fig. 5C).

**Figure 7:**
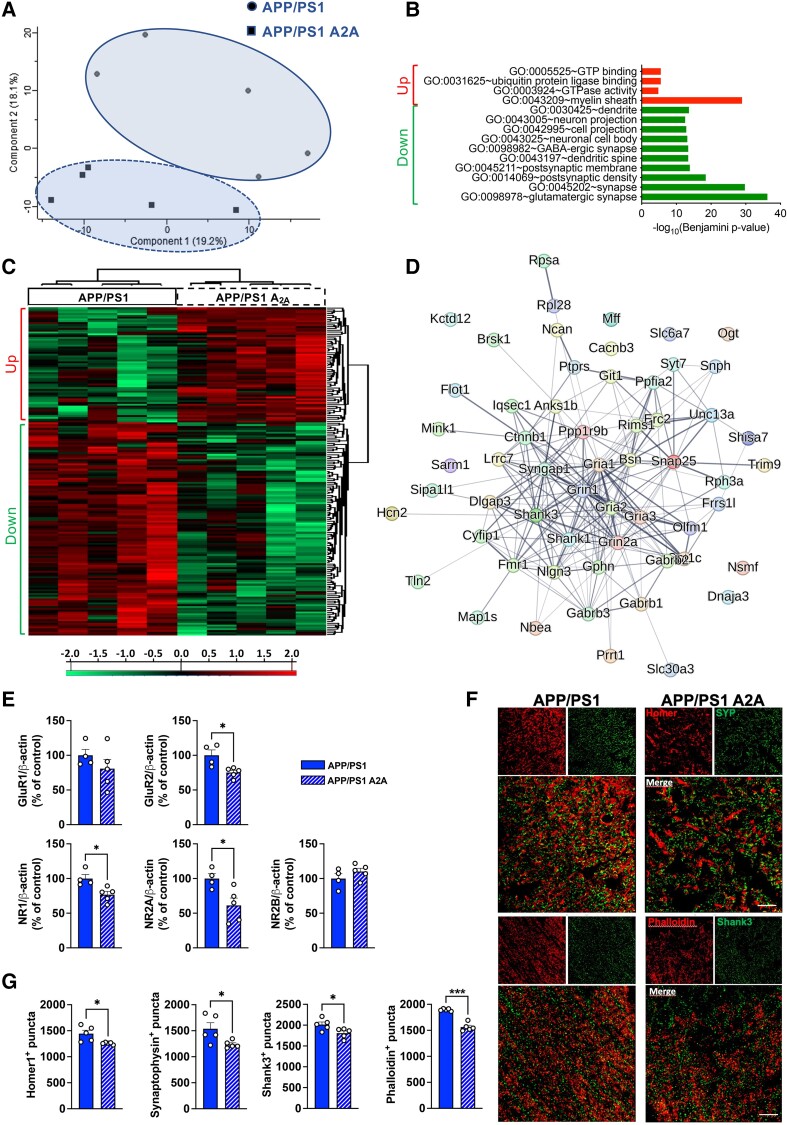
**Neuronal upregulation of A_2A_ receptors promotes loss of synapses in the hippocampus of APP/PS1 mice.** (**A**–**D**) MS-based proteomics analysis of the hippocampus of APP/PS1 A2A versus APP/PS1 animals at the age of 6 months (*n* = 5 per group). (**A**) Principal component analysis (PCA) from proteomics data of hippocampal samples of APP/PS1 and APP/PS1 A2A animals. *n* = 5 mice per group. The first and second principal components explained 19.2% and 18.1% of the variance, respectively. (**B**) Heat map representation of hierarchical clustering to display the results of a *t*-test statistical analysis comparing the levels of protein expression in two groups, APP/PS1 and APPP/PS1 A2A, for proteins that were differentially expressed (*P* < 0.05). (**C**) Functional annotation of the 62 overexpressed proteins (red) related to myelin sheath and 115 underexpressed proteins related to neurons and synapses (green) in APP/PS1 A2A versus APP/PS1 A2A was performed with the Database for Annotation, Visualization and Integrated Discovery (DAVID) for the GO term ‘Biological Process’. (**D**) Known and predicted protein interaction (STRING) of the downregulated genes belonging to the significant GO term ‘synapse’ shown in **C**. (**E**–**G**) Quantification of synaptic proteins. (**E**) Quantification of GluR1, GluR2, NR1, NR2A and NR2B levels between APP/PS1 A2A and APP/PS1 mice using western blot. Analysis revealed a significant decrease in GluR2, NR1 and NR2A in the hippocampus of APP/PS1 A2A compared with APP/PS1 mice. *P < 0.05, Student’s *t*-test. *n* = 4–5 mice per group. (**F**) Representative images from Airyscan confocal microscopy of presynaptic (synaptophysin, SYP, green) and postsynaptic (Homer1, red) markers (*top*) as well as postsynaptic marker Shank3 (green) and phalloidin (F-actin staining, red) (*bottom*) in the hippocampus of APP/PS1 and APP/PS1 A2A mice (scale bar = 10 µm). (**G**) Significant decreases in Homer1^+^, synaptophysin^+^, Shank3^+^ and phalloidin^+^ puncta were found in the CA1 area of APP/PS1 A2A versus APP/PS1 mice. *P < 0.05 and ***P < 0.001, Student’s *t*-test. *n* = 5 mice per group.

This unsupervised analysis also brought about two additional observations. When we compared gene expression from modules M4 (Supplementary Fig. 10A) to our previous results obtained in our tauopathy mouse model, Thy-Tau22 mice (RNA-seq data from Carvalho *et al*.^27^), we observed the same effect as in APP/PS1 mice, i.e. no effect of the presence of A_2A_R but synergy with the presence of either APP/PS1 or Tau22 genotypes on synaptic pathways^27^ (Figs 5B and 7, proteomic data). Conversely, the module M7 identified a set of genes that remained unaltered in APP/PS1 or A_2A_R mice but were upregulated in APP/PS1 A2A animals (Supplementary Fig. 10B) with an opposite effect observed in Thy-Tau22 mice. Pathway analysis highlighted that these genes are downregulated in the hippocampus of Huntington’s disease mouse models (Supplementary Fig. 10B). They do not associate significantly with specific biological functions but some are neuronal genes associated to the postsynaptic compartment (*Gabrd*, *Htr7*, *Kcnj4*, *Oprk1* and *Tacr1*).

### Proteomic signature associated with the neuronal overexpression of A_2A_R in APP/PS1 mice

In addition to transcriptomics, we performed a proteomic evaluation of hippocampal changes occurring in APP/PS1 A2A versus APP mice. Five samples per group were included with a PCA analysis (Fig. 7A). A heatmap representation of hierarchical clustering was used to display the differentially expressed proteins between APP/PS1 A2A and APP/PS1 samples (*P* < 0.05; Fig. 7B, Up: over-expression and Down: under-expression). A total of 177 proteins were found to be differentially expressed with a |Log2 fold-change| of at least 0.32, 115 proteins being underexpressed and 62 proteins overexpressed in the hippocampus of APP/PS1 A2A animals (Supplementary Table 4). None of the corresponding mRNAs was found to be significantly altered per the transcriptomic analysis (not shown). Functional biological process annotations (Fig. 7C) indicated that upregulated proteins in APP/PS1 A2A mice showed particular enrichment in proteins related to myelin sheath. Strikingly, the downregulated proteins were related to neurons and glutamatergic synapses (Fig. 7C). Using the STRING database, we observed a strong interaction between the 58 members of the ‘synapse’ (GO:0045202) cluster, including for instance, Gria2, Grin1, Grin2a or Shank3 (Fig. 7D). We further assessed the predicted role of the 115 downregulated proteins in the synaptic compartment using SynGO ontologies and annotations (Supplementary Fig. 11A).^45^ We observed that most of the synaptic proteins annotated were related to synaptic assembly, postsynaptic-structures, fusion of synaptic vesicles or synaptic transmission.

Downregulation of synaptic proteins was further validated using biochemical and immunohistochemical approaches. As shown in Fig. 7E, we observed the downregulation of GluR2 [*t*(7) = 3.193, *P* = 0.015], NR1 [*t*(7) = 3.176, *P* = 0.015] and NR2A [*t*(7) = 2.874, *P* = 0.023; Student’s *t*-test] in APP/PS1 A2A versus APP/PS1 mice using western blot, as well as the downregulation of Shank3 [*t*(8) = 2.514, *P* = 0.036; Student’s *t*-test] using high-resolution confocal microscopy (Fig. 7F and G). Accordingly, we observed a significant loss of phalloidin (F-actin staining) [*t*(8) = 9.155, *P* < 0.0001], Homer1 [*t*(8) = 2.852, *P* = 0.021] and synaptophysin [*t*(8) = 2.467, *P* = 0.038; Student’s *t*-test] in the CA1 area of APP/PS1 A2A versus APP/PS1 mice (Fig. 7F). In contrast, VGAT, a marker of inhibitory synapses, remained unaffected (*P* > 0.05; not shown). Such loss of synapses was neither associated with changes in the expression of microglial genes linking neuronal A_2A_R upregulation to tau-induced synapse loss such as *C1qa*, *Pycard* and *Csf1r* (Supplementary Fig. 12A) nor with the engulfment of synapses by microglial cells (Supplementary Fig. 12B), suggesting that the loss of hippocampal synapses and associated impaired memory found in APP/PS1 A2A mice is not subsequent to a microglial-based pruning as we previously observed in a tauopathy context.

## Discussion

Considering the prime role of A_2A_R in synaptic fine-tuning,^5,11^ the aberrant plasticity changes associated with its neuronal dysregulation in aged and AD conditions^21^ as well as the beneficial impact of its pharmacological or genetic blockade against memory and hippocampal plasticity impairments in models of amyloidogenesis or amyloid toxicity,^5,13-15,17-21^ we hypothesized that pathological neuronal upregulation of A_2A_R might favour AD lesion development and synaptic alterations promoted by amyloid pathology.

To tackle this question, we crossed a new transgenic Tet-off mouse model (TRE-A2A)^27^ with APP/PS1 mice with litters intrahippocampally injected with a viral vector allowing the expression of the tTA transactivator to elicit neuronal A_2A_R overexpression. Animals were injected at the age of 3 months and evaluated 3 months later (i.e. at 6 months of age), a time point at which APP/PS1 mice normally exhibit ongoing amyloid pathology but no memory deficits.^39,40^ Strikingly, we observed, using Y-maze and Barnes maze, that at such an early time point, spatial memory was strongly impaired in APP/PS1 mice overexpressing neuronal A_2A_R—as it is expected to occur at later time points in APP/PS1 mice—when compared with the other littermate groups (WT, A2A and APP/PS1), which exhibited proper memory abilities. Therefore, early upregulation of A_2A_R in a hippocampal environment of ongoing amyloid pathology worsens the behavioural phenotype of APP/PS1 mice. These data are in line with a previous patch-clamp study showing that neuronal A_2A_R downregulation driven by shRNA interference restores hippocampal plasticity in APP/PS1 mice at an early pathological stage.^15^

Using supervised and unsupervised analysis of our transcriptomic data, we unveiled two types of responses arising from A_2A_R neuronal upregulation in APP/PS1 mice. First, we showed an exacerbation of the microglial impairments naturally occurring in APP/PS1 mice following A_2A_R neuronal upregulation. Interestingly, genes associated with module 9 were also associated with the pathological evolution of APP/PS1 mice as well as changes occurring in the hippocampus of AD patients. None of the genes found modified by A_2A_R was related to AD genetic susceptibility variants previously associated with AD risk in genome-wide association studies and largely expressed by microglia (not shown).^46^ However, several of the genes exclusively changed in APP/PS1 A2A mice (*Ccl4*, *Cd72*, *Cd84*, *Pdcd1*, *Sclc11a1*, *St14* and *Tlr2*) belonged to the DAM (disease-associated microglia) signature associated with neurodegeneration in another amyloid model.^47^ In agreement with transcriptomics, although peri-plaque microglia remained unaffected by the neuronal overexpression of A_2A_R, the overall parenchymal staining for Iba1^+^ microglia was, however, significantly enhanced in APP/PS1 A2A mice together with a reduced process complexity, supporting an activation of microglial cells in the hippocampus. Resulting neuroinflammation may likely explain synapse loss and cognitive deficits.^48^ Interestingly, in a previous study evaluating the consequences of neuronal overexpression of A_2A_R in a tauopathy model, the transcriptomic analysis also uncovered a microglial-related response.^27^ However, despite the upregulation of microglial-selective genes in both APP/PS1 and tau transgenic mice overexpressing A_2A_R, no overlap could be observed. In sharp contrast to the observations made in APP/PS1 mice, A_2A_R-related changes in tau mice were not associated with morphological changes linked to microglial activation but rather with processes related to synaptic pruning.^27^

Moreover, we uncovered two gene modules corresponding to changes never observed in A_2A_R and APP/PS1 mice at this pathological stage but that are triggered in APP/PS1 A2A mice and are related to mitochondria- and synapse-related pathways. Regarding mitochondria, the comparison with scRNA-seq data particularly emphasized that mitochondrial impairments occurred in neurons. Again, mitochondrial gene impairments were associated with the pathological evolution of APP/PS1 mice as well as AD patients. Such mitochondrial impairment is likely to be involved in the reduction of energy production that favours synaptic loss.^49^ Puzzlingly, previous data obtained in a model of Niemann-Pick disease indicated that activation of A_2A_R rescues compromised mitochondrial functionality (mitochondrial inner membrane potential and expression of complex IV of the mitochondrial respiratory chain) in line with other studies.^50,51^ Mechanisms linking this neuronal-autonomous effect of A_2A_R upregulation that negatively impacts mitochondrial function will therefore deserve further studies.

Synaptic changes seen using transcriptomics are in agreement with our proteomic data and were validated by biochemistry and immunohistochemistry. Moreover, our data highlight a greater susceptibility of excitatory over inhibitory (VGAT) synapses, in agreement with our previous work using tau transgenic mice.^27^ Taken together, these data suggest that the pathological upsurge of A_2A_R in neurons might render excitatory synapses particularly vulnerable to amyloid, in agreement with previous data showing that pharmacological or constitutive deletion of A_2A_R reduces the *in vitro* and *in vivo* acute synaptotoxicity of Aβ_1–42_.^14^ While the overall synaptic/memory outcomes found in APP/PS1 A2A mice were similar to those previously demonstrated in Tau A2A animals,^27^ the loss of synapses seen in the former was not mediated by a C1q-dependent phagocytosis by microglial cells, as attested by the lack of C1q expression changes and the absence of internalization of synaptic material into microglial lysosomes.

It is also interesting to mention that unsupervised analysis uncovered a module with genes associated with Huntington’s disease that co-varied the same way as in Tau A2A mice (i.e. downregulation), whereas they showed an opposite variation in APP/PS1 A2A mice (i.e. upregulation). The significance remains unclear. However, it is interesting to note that, (i) from the memory point of view, A_2A_R blockade provides the same beneficial outcome in Huntington’s disease and tau transgenic models^16,52^; and (ii) that Huntington’s disease has been associated with the development of tau pathology.^53,54^

Several mechanisms other than microglial neuro-inflammation or mitochondrial defects could underlie this A_2A_R-mediated synaptic loss in APP/PS1 A2A mice. An initial possibility could be related to a plausible impact of A_2A_R upregulation on amyloid burden. However, while western blot analysis indicated an increase in total Aβ species (6E10-immunoreactive), Aβ_42_, Aβ_42_/Aβ_40_ ratio and APP processing as well as plaque load and plaque size distribution (not shown) remained unaltered in APP/PS1 A2A mice. This slight impact of neuronal A_2A_R upregulation on amyloidogenesis was unexpected, considering the significant reduction of amyloid plaques and Aβ_42_/Aβ_40_ ratio previously observed following the chronic delivery of an A_2A_R antagonist in the same mouse model^17^ as well as the reduced accumulation of Aβ in the brain of APP mice and aged individuals induced by caffeine, a non-selective A_2A_R antagonist.^7,55,56^ One explanation for such an apparent discrepancy could be linked to a non-neuronal impact of A_2A_R antagonists and the involvement of receptors expressed by other cells, possibly astrocytes.^18,26,57^ While the overall parenchymal level of p-tau remained unaltered in APP/PS1 A2A mice, we found a significant increase in p-tau around amyloid plaques, suggesting that neuronal upregulation of A_2A_R favours neuritic tau pathology. Interestingly, neuritic plaques, which have been associated with synaptic loss and cognitive decline in AD patients,^58,59^ are sites of Aβ–tau interaction^58,60^ and are thought to provide a microenvironment that facilitates the seeding and expansion of tau pathology and, hence, AD progression.^61,62^ In an amyloid context, A_2A_R neuronal dysregulation would therefore favour Aβ–tau interaction at neuritic plaques, the loss of synapses and, ultimately, the development of cognitive deficits. Recent data support the idea that microglia would restrain the development of p-tau at neuritic plaques.^53,63^ However, we could not detect any significant microglial changes around the plaques. Therefore, at present, the mechanism by which neuronal A_2A_R upregulation favours the development of p-tau around amyloid plaques remains elusive and will deserve further attention in the future.

Neuronal A_2A_ upregulation, therefore, accelerates synaptic loss and memory impairment within an amyloid burden context, rendering the hippocampal glutamatergic synapses particularly vulnerable. Overall, A_2A_R neuronal upregulation promotes different changes at the microglial level, presumably due to different neuroglial signalling depending on the amyloid or the tau context, ultimately converging to similar synaptic and memory outcomes. The neuroglial signalling and molecular mechanisms at play warrant further investigations. Finally, considering that amyloid but also tau positivity are found in cognitively unimpaired individuals in the elderly, from the present data and our previous observations in tau mice,^27^ it is conceivable that early neuronal upregulation of A_2A_R might play a prime role in the development of cognitive deficits in aged individuals and in the conversion to AD. Taken together, our present and previous data support that the clinical repurposing of A_2A_R antagonists such as Istradefylline would be of clinical interest in prodromal AD patients.

## Supplementary Material

awae113_Supplementary_Data

## Data Availability

The data that support the findings of this study are available from the corresponding author upon reasonable request.
