# Supplementary material for: Neuronal A2A receptor exacerbates synapse loss and memory deficits in APP/PS1 mice
Source: Brain. 2024 Jul 5;147(8):2691–705. doi: 10.1093/brain/awae113 (PMC11292904; doi:10.1093/brain/awae113)
Supplement: awae113_Supplementary_Data [file awae113_supplementary_data.zip › brain-2023-02499-File013.pdf]

Supplementary material and methods

Behavioral analysis. Behavioral experiments were conducted at an early stage, between 5 and 6 months of age, before memory impairments are present in the APP/PS1 mouse model. Mice were randomly assigned by experimenters blinded to the genotype and experiments performed per procedures given as follows:

-*Actimetry.* Mice were placed in the center of an infrared Actimeter (45x45x35cm; Bioseb), composed by a 2-dimensional square frame, and left for 10 min. Spontaneous behavior of mice was tracked with velocity and distance moved recorded by Actitrack software (Bioseb).

-*Elevated Plus Maze.* The elevated plus maze was used to investigate anxiety-related behavior. The apparatus consists of a plus-shaped maze with two closed and two open arms (30 cm long x 6.5 cm wide). Mice were placed at the center of the maze with their face in the direction of a closed arm and were allowed to explore freely for 5 min. Time spent in open arms was tracked using the Ethovision XT tracking system (Noldus Information Technology, Wageningen, The Netherlands).

-*Y-maze task.* Short-term spatial memory was assessed in a spontaneous novelty-based spatial preference Y-maze test. All arms of the Y-maze were 28 cm long, 6.2 cm wide, with 15-cm-high white opaque walls. Different extra maze cues were placed on the surrounding walls. To avoid intra-maze odor cues, sawdust was placed in the maze and mixed between each phase. Allocation of arms was counterbalanced within each group. During the learning phase, mice were placed at the end of the 'start' arm and were allowed to explore the 'start' arm and the 'other (familiar)' arm for 5 min (beginning from the time the mouse first left the start arm). Access to the third arm of the maze ('novel' arm) was blocked by an opaque door. The mouse was then removed from the maze and returned to its home cage for 2 min. In the test phase the mouse was placed again in the 'start' arm of the maze, the door of the 'novel' arm was removed, and mouse behavior recorded for one minute (from the time the mouse first left the start arm). The amount of time the mouse spent in each arm of the maze was recorded during both learning and test phases using EthovisionXT (Noldus). For the learning phase, we calculated the percentage of time spent in the 'other' (familiar) vs. the start arm. For the test phase, we calculated the percentage of time spent in the familiar (F) arm vs. the 'novel' (N) arm.

-*Barnes Maze Task.* Spatial learning and memory were evaluated using the Barnes maze task. This maze is a white circular PVC open platform surface (120 cm of diameter) with 40 equally spaced holes (5 cm of diameter) located at 5 cm from its circumference and a black escape box

located under one of the 40 holes. The platform is placed on a swivel system (to rotate it easily) in the center of the room, elevated 80 cm above the floor, enlighten by a flood light (800 lux) placed above and surrounded by spatial cues. Mice completed an habituation trial to become familiar with the maze environment and to practice descending into the escape box. Mice were free for 5 min to explore the area around the escape hole and the escape box. Then, mice completed 4 days of acquisition training with 4 trials per day. For each trial, mice were placed in the start tube in the center of the maze (for 5-10 s) and then trained to locate the escape hole (randomized for all mice) using spatial cues surrounding the maze. If a mouse did not enter the escape hole in 3 min, it was gently guided to the escape hole. Mice remained 60s in the escape box before returned to its home cage. The inter-trial interval was 15 min. To reduce intra-maze odor cues, the maze surface and escape box were cleaned with 70% ethanol between each trial and the maze was rotated clockwise a quarter turn every day. For each trial, path length to enter the escape box was recorded using the Ethovision XT tracking system (Noldus). 24h after the last day of the training, mice completed a 2-min probe trial where the escape box was removed. We calculated, on the one side, the percentage of time spent in the target quadrant (T) versus the averaged percentage of time spent in all the othet quadrants (O) i.e. the average of the percentage spent in the two adjacent and the opposite quadrants.

Biochemical analyses. Protein concentrations were quantified using the BCA assay (Pierce), and samples diluted with lithium dodecyl sulphate buffer supplemented with reducing agents (Invitrogen) and then separated on 4–12% NuPAGE Novex or 12% Criterion Bis-Tris Gels gels (Invitrogen). Proteins were transferred to nitrocellulose or PVDF membranes, which were then saturated with 5% non-fat dried milk or 5% bovine serum albumin in TNT (Tris 15mM pH 8, NaCl 140mM, 0.05% Tween) and incubated at 4°C for 24h with the primary antibodies (**Supplementary Table 1**). Appropriate HRP-conjugated secondary antibodies (anti-mouse PI-2000 and anti-rabbit PI-1000, Vector Laboratories; anti-guinea pig A7289, Sigma-Aldrich) were incubated for 1h at RT and signal was visualized using a chemoluminescence kit (ECL, Amersham Bioscience) and a LAS4000 imaging system (Fujifilm). Results were normalized to actin or GAPDH and quantification was performed using ImageJ software.

Protein preparation for proteomic analysis. 50 µg of hippocampal proteins were collected and reduced with the equivalent volume of 0.1 M dithiothreitol for 15 min at 95°C. Filter-aided sample preparation (FASP)30 was performed using Amicon 30K centrifugal filters (Millipore) and trypsin (40 µg/ml in 50 mM ammonium bicarbonate, 37°C, overnight) for digestion of the

reduced protein extracts. The digests were collected by centrifugation and the filter devices were rinsed with 0.5M NaCl. The digestion was stopped by adding 5% TFA to the digests. Collected samples were desalted using C18 ZipTip device (Millipore), dried under vacuum and reconstituted in 0.1% Formic acid/ACN (98:2, v/v) before NanoLC-MS/MS analysis.

ELISA measurements. Hippocampal levels of human $A\beta_{1-40}$, $A\beta_{1-42}$ and $A\beta_o$ (oligomers) were measured using ELISA kits (Invitrogen, CA, USA; IBL-International, Hamburg, Germany or IBL 82E1-specific assay) following manufacturer's instructions and as described.[17] Briefly, 20 µg of proteins from the above-mentioned Tris sucrose homogenates were diluted in Guanidine/Tris buffer (Guanidine HCl 5M and Tris 50 mM pH 8), sonicated and incubated for 1h at 4°C under agitation. Samples were then diluted in a BSAT-DPBS solution (KCl, KH_2PO_4, NaCl, Na_2HPO_4, BSA 5%, Tween-20 0.03% pH 7.4). The homogenates were centrifuged at 12000 g for 15 min at 4°C. Supernatants were collected for the analysis of $A\beta_{1-40}$ and $A\beta_{1-42}$ by colorimetric immunoassays. Absorbance was measured by Multiskan Ascent counter (ThermoLab Systems). The normalized amounts of Aβ were expressed as pg/mL.

Regarding tau ELISA, Tris/Sucrose homogenates were prepared at 1mg/mL in RIPA buffer (Tris 100mM, NaCl 300mM, SDS 0.2%, sodium deoxycholate 1% and NP-40 2%, pH 8) and quickly sonicated. For pS199-tau mouse ELISA, steps were performed according to the manufacturer's instructions (Invitrogen). For total tau ELISA, we developed a homemade test with new monoclonal antibodies developed in the lab. For capture, we coated plates (Maxisorp F8, Nunc) with an anti-tau 9H12 antibody against aas 162–175[29] overnight in carbonate buffer (NaHCO3 0.1M with Na2CO3 0.1M, pH9.6). After 5 washes, plates were blocked in PBS containing 2% casein during 1h. We used recombinant tau (1N4R) for the standard curve (0.039 to 200ng/mL). Samples were diluted at 1:120 and incubated overnight with tau C-terminal detection antibody 7F5 against aas 427-441. After 5 washes, the assay was then incubated with an anti-IgG1 HRP conjugated antibody (Southern Biotech). After 1h, the complexes were revealed with substrate (Tetramethyl benzidine (Sigma-Aldrich) in citrate/phosphate buffer during 30 min. The reaction was stopped by addition of sulfuric acid changing the color from blue to yellow. Absorbance was measured with a spectrophotometer (Multiskan FC, Thermo) at 450 nm. Interpolation of the standard curve was performed using GraphPad Prism Software.

mRNA extraction and quantitative real-time RT-PCR analysis. One microgram of total RNA was reverse-transcribed using the Applied Biosystems High-Capacity cDNA reverse transcription kit. Quantitative real-time RT-PCR analysis was performed on an Applied

Biosystems Prism 7900 System using Taqman probes. The thermal cycler conditions were as follows: hold for 10 min at 95°C, followed by 45 cycles of a two-step PCR consisting of a 95°C step for 15 s followed by a 60°C step for 25 s. Sequences of Taqman probes used are given in **Supplementary Table 2**. Cyclophilin A was used as internal control. Amplifications were carried out in triplicate and the relative expression of target genes was determined by the ΔΔCT method.

Histological processing. Antibodies used in this study are listed in **Table S1**. For 6E10 immunohistochemistry, sections were pretreated with 80% formic acid for 3 min and were permeabilized with 0.2% Triton X-100/Na-PB. Sections were then blocked with 10% "Mouse On Mouse" Kit serum (Vector laboratories) for 1h before incubation with mouse biotinylated anti-Aβ antibody (6E10) at 4°C overnight. After washing in PBS, the sections were incubated with the ABC kit (Vector laboratories) for 2 h and developed using 3,30-diaminobenzidine as the peroxidase substrate (DAB, Sigma). For $A_{2A}R$ and Iba1 immunostainings, sections were permeabilized with 0.2% Triton X-100/Na-PB and then blocked with 10% Normal goat serum (Vector laboratories) for 1h before incubation with primary antibodies at 4°C overnight. Biotinylated-conjugated secondary antibodies (1/500, Vector) were incubated for 1h at room temperature. Revelation was performed using ABC method (Vector Laboratories) with DAB (Sigma). For immunofluorescent stainings, brain sections were washed with Na-PB buffer and permeabilized with 0.2% Triton X-100/Na-PB and then blocked with the appropriate normal serum: donkey serum (1/100; Sigma), goat serum (1/100; Vector laboratories) or "Mouse On Mouse" Kit serum (1/100; Vector laboratories). Brain sections were then incubated with primary antibodies overnight at 4°C. Appropriate Alexa Fluor488-, Alexa Fluor568- and Alexa Fluor647-conjugated secondary antibodies (1:500, Life Technologies) were incubated for 1h at room temperature. For phalloidin staining, sections were incubated with the dye for 30 min at RT. For Thioflavin staining, sections were incubated with the dye for 30 min at RT, then washed with successive baths of ethanol (70% then 95% v/v). Sections were counterstained with DAPI (1/5000, 62248, Thermoscientific) for 10 min and treated for 10 min in 0.3% (v/v) Soudan Black (Sigma-Aldrich) with 70 % ethanol to block autofluorescence. Slices were mounted with Dako Fluorescent mounting medium.

Image acquisition and image analysis. Stainings were quantified from 6-7 sections per animal for immunhistochemistry and 2-3 sections for immunofluorescence. For 6E10, $A_{2A}R$ and Iba1 staining, images were acquired using 20x objective Zeiss Axioscan Z1 slide scanner.

Quantification of the 6E10 staining intensity was performed using Mercator software (Explora Nova, USA). Hippocampal Aβ plaque load expressed as the percentage of 6E10 stained area was calculated. Quantification of Iba1 positive area was performed with Image J software (Scion Software). For immunofluorescence, images were acquired using a 40x oil objective Zeiss LSM-710 confocal laser-scanning microscope to define co-localization of $A_{2A}R$ with neuronal vs. glial markers. 3D reconstruction of 2D confocal image stacks was performed using Imaris software (Bitplane, USA). For the 3D morphological analysis of microglia, regions of the hippocampal CA1 area were imaged using a Zeiss Spinning Disk high-resolution microscope taking-up to 15 z-stacks at 1 um steps with a 40x oil objective. The $Iba1^+$ microglia were 3D-reconstructed using the “Surface” and “Filament Tracer” rendering functions in Imaris and the microglial morphology was studied using sholl analysis. This analysis was done at distance from the amyoid plaques (>50µm). Sholl intersections are the number of times a cell extension in a 2D representation intersects the concentric circles, or radius, positioned at an interval of 1µm and starting from the cell soma.

For Homer1, Synaptophysin, Shank3 and Phalloidin single-plane images (3 regions of interest -ROIs- per section) were acquired on an Zeiss LSM-710 Airyscan confocal microscope with a 63x oil objective. Quantification of number of puncta for each marker was performed using the « Spots » function in Imaris. Spots were detected for each channel using an x-y-z size of 0,4 µm and automated background subtraction. For microglial engulfment analysis of synapses, hippocampal CA1 regions were imaged on a Zeiss LSM-710 Airyscan microscope taking-up to 43 z-stacks at 0.16 µm steps with a 63x oil objective. Cd68-positive lysosomes were 3D-reconstructed using the « Surface » rendering function in Imaris and Synaptophysin and Homer1 puncta (reconstructed with « Spots ») inside the $Cd68^+$ lysosomes were quantified. Quantification of AT8-positive area surrounding amyloid plaques within a 15 µm distance was also performed on Imaris to create surface of each section based on the threshold applied to all images (2-3 ROIs per section). To do so, an average of 6-7 images per sections of AT8 and $Thioflavin^+$ plaques was acquired with a 63x oil objective on Zeiss LSM-710.
