# Supplementary material for: Neuronal A2A receptor exacerbates synapse loss and memory deficits in APP/PS1 mice
Source: Brain. 2024 Jul 5;147(8):2691–705. doi: 10.1093/brain/awae113 (PMC11292904; doi:10.1093/brain/awae113)
Supplement: awae113_Supplementary_Data [file awae113_supplementary_data.zip › brain-2023-02499-File014.pdf]

Supplementary Figures

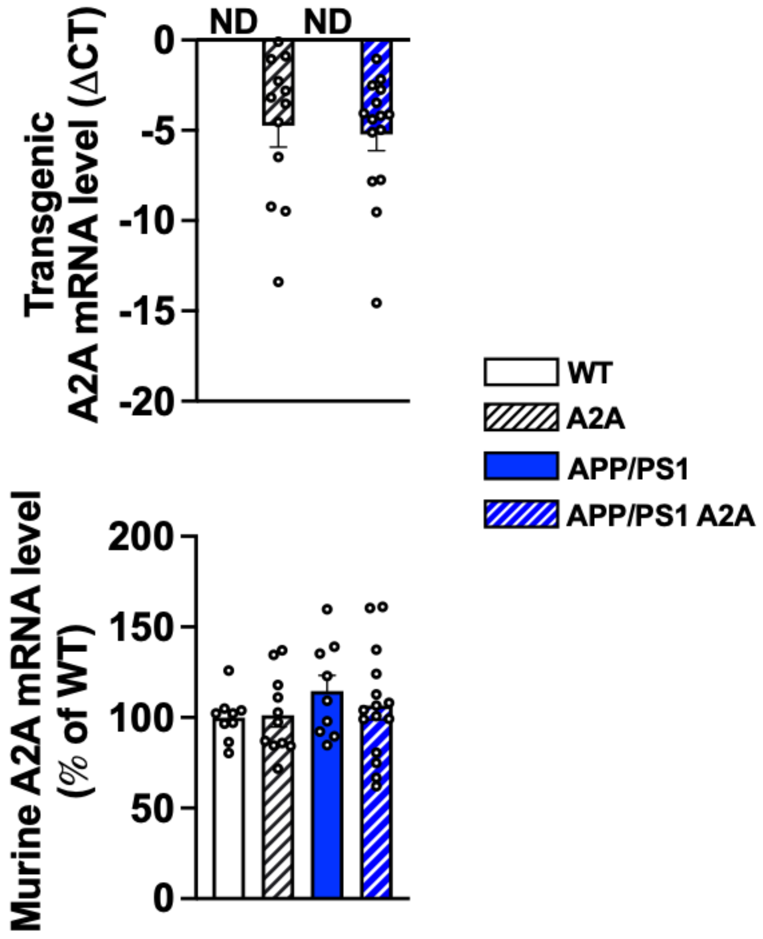

Supplementary Figure 1. Levels of the transgenic (upper graph) and endogenous A2A (lower graph) mRNAs in the different experimental groups.

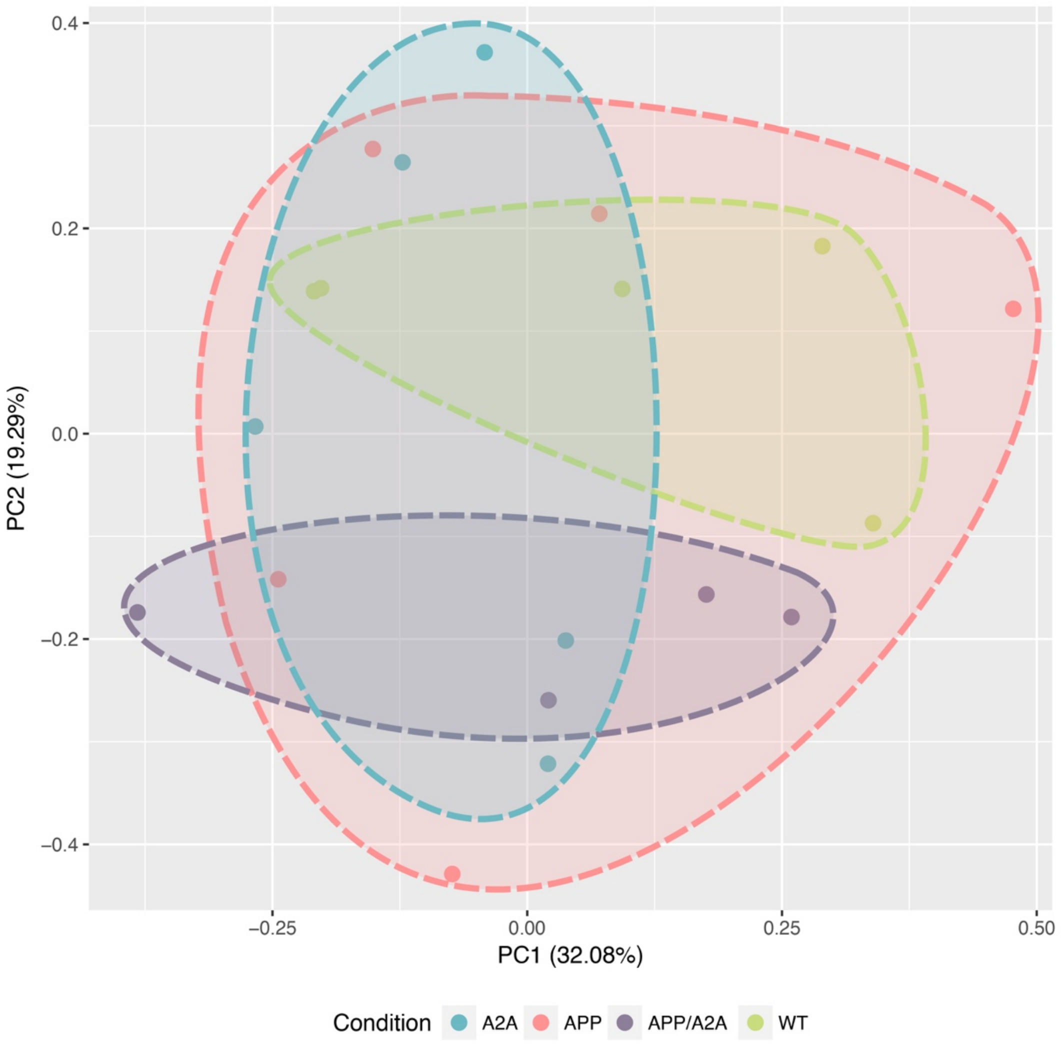

Supplementary Figure 2. Transcriptome profiling. Principal Component Analysis (PCA) performed from hippocampal transcriptomics from WT, A2A, APP/PS1 and APP/PS1 A2A animals. Dots represent samples and are colored according to the genotype. The first and second principal components explained 32.08% and 19.29% of the variance, respectively.

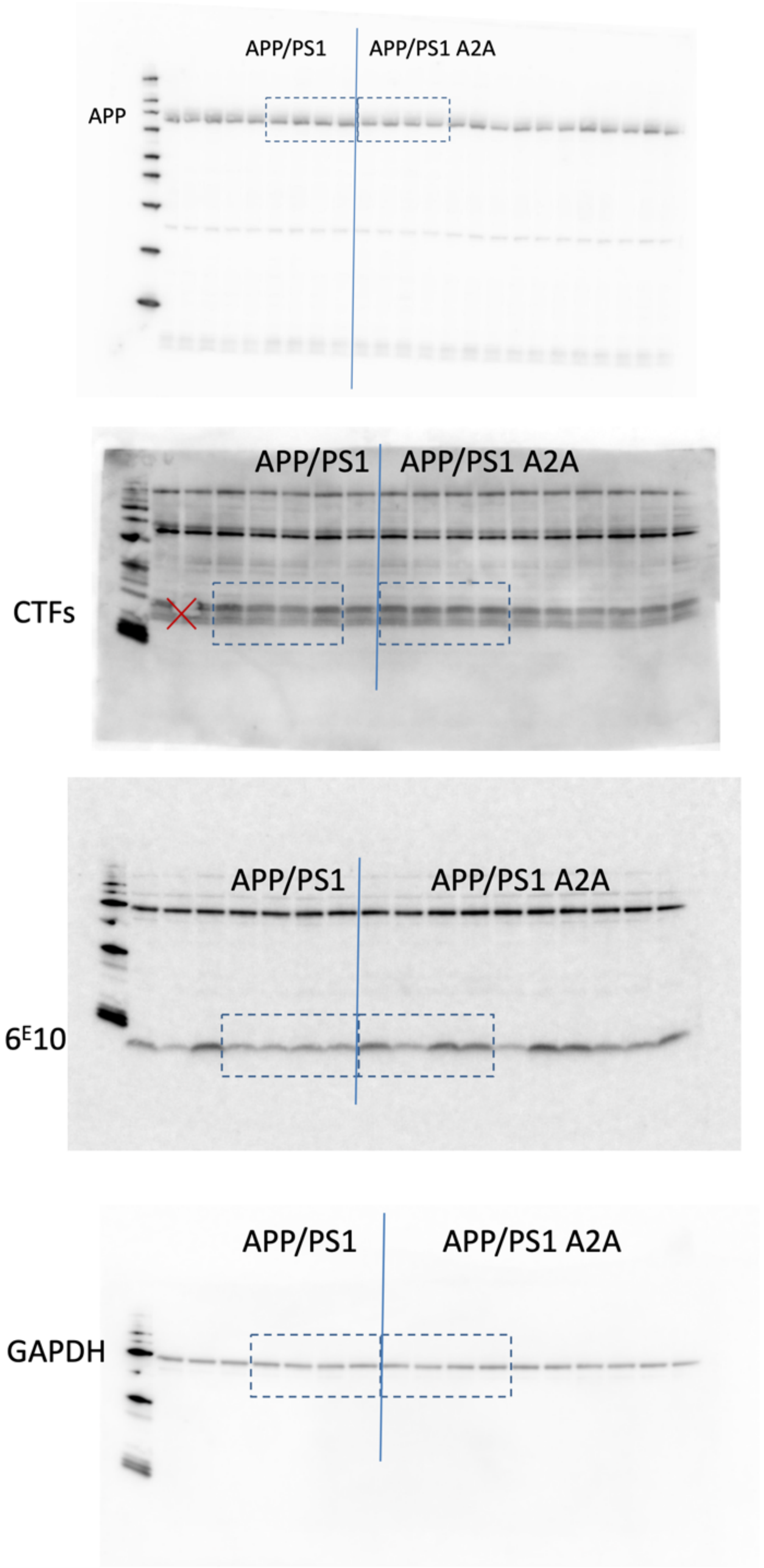

Supplementary Figure 3. Original western blots of the Figure 3F.

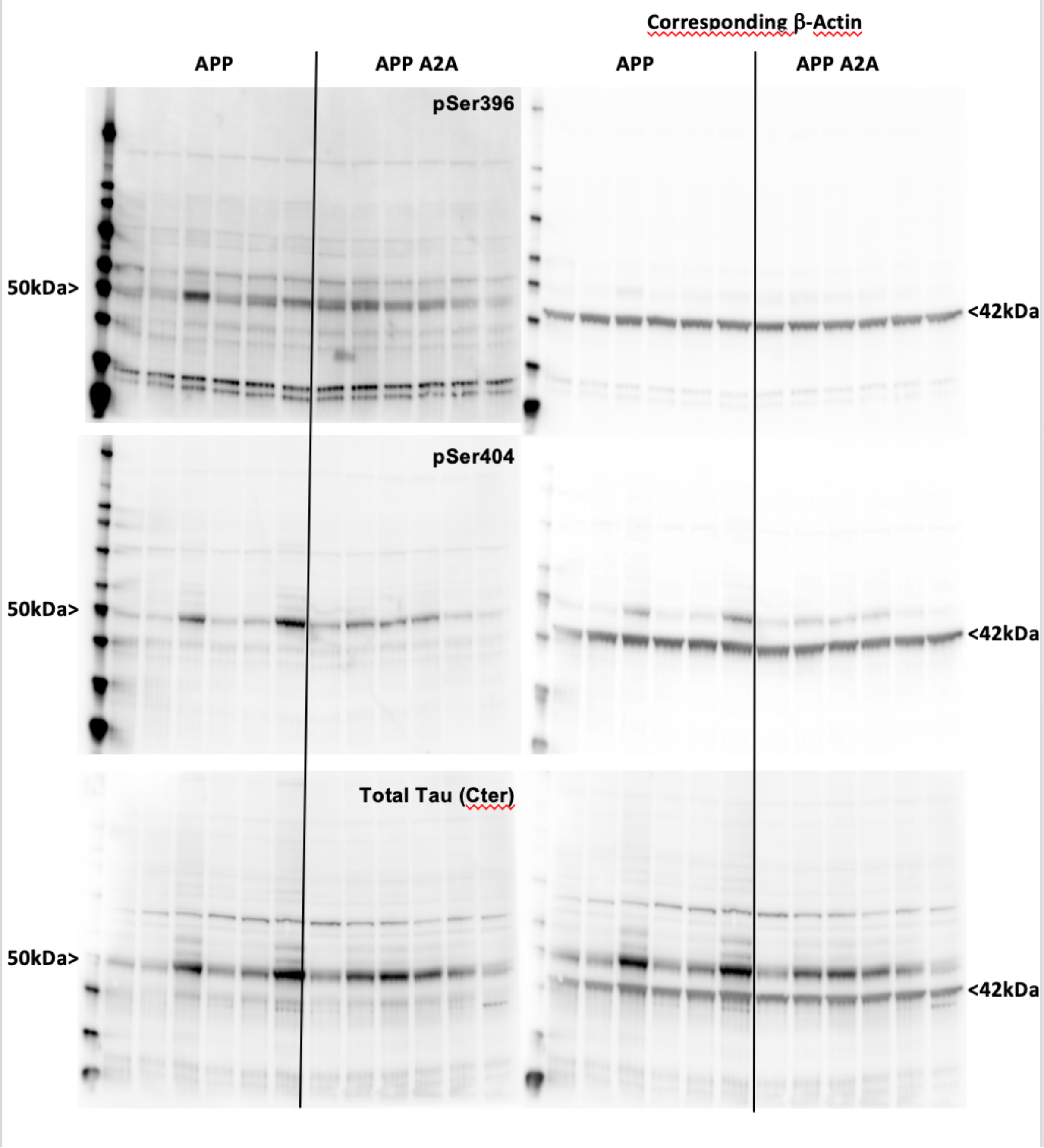

Supplementary Figure 4. Original western blots of the quantification on Figure 3G.

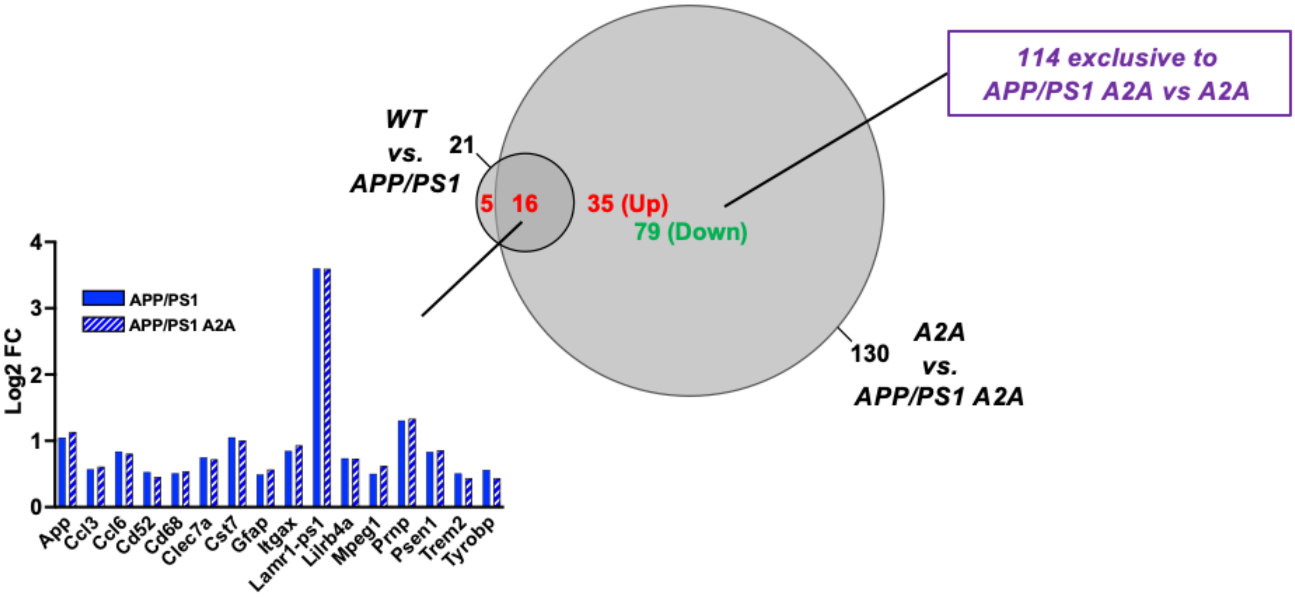

Supplementary Figure 5. Analysis of differentially expressed genes (DEGs; |Log2 fold change|>0.32 and adjusted P-value<0.05) in the hippocampus of WT, A2A, APP/PS1 and APP/PS1 A2A animals at the age of 6 months (N=4-5 mice per group). Venn diagrams shows the number of DEGs between APP/PS1 vs. WT mice (21 upregulated genes, red) as well as APP/PS1 A2A vs. A2A animals (51 upregulated, red; and 79 downregulated genes, green). The 16 DEGs found commonly upregulated in APP/PS1 mice vs. WT and APP/PS1 A2A mice vs. A2A, were, for most of them being related to neuroinflammatory processes. 114 genes were exclusive to APP/PS1 A2A mice.

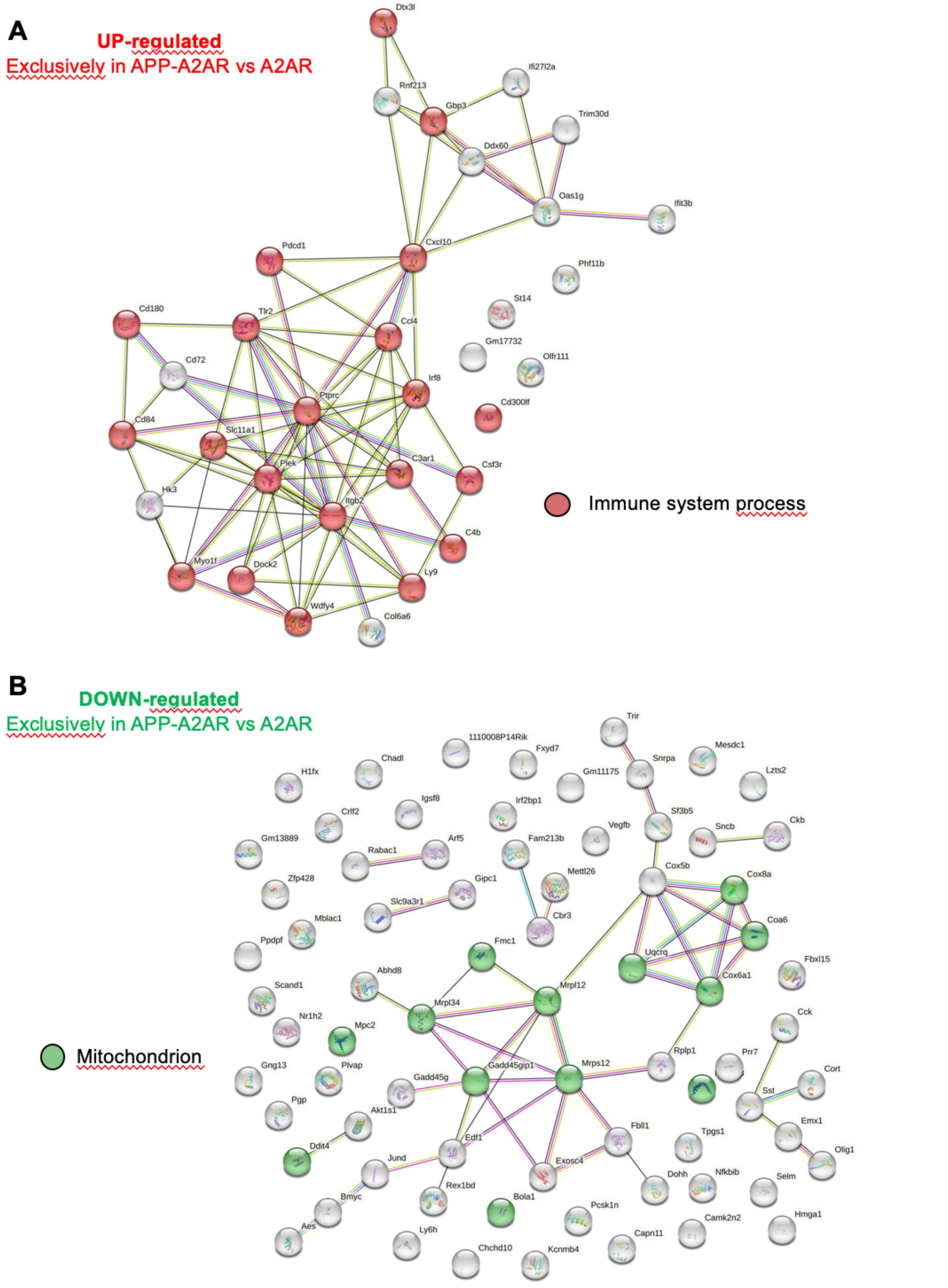

Supplementary Figure 6. STRING network analysis of the exclusive 35 increased (**A**) and 79 decreased (**B**) genes exclusive to APP/PS1 A2A mice.

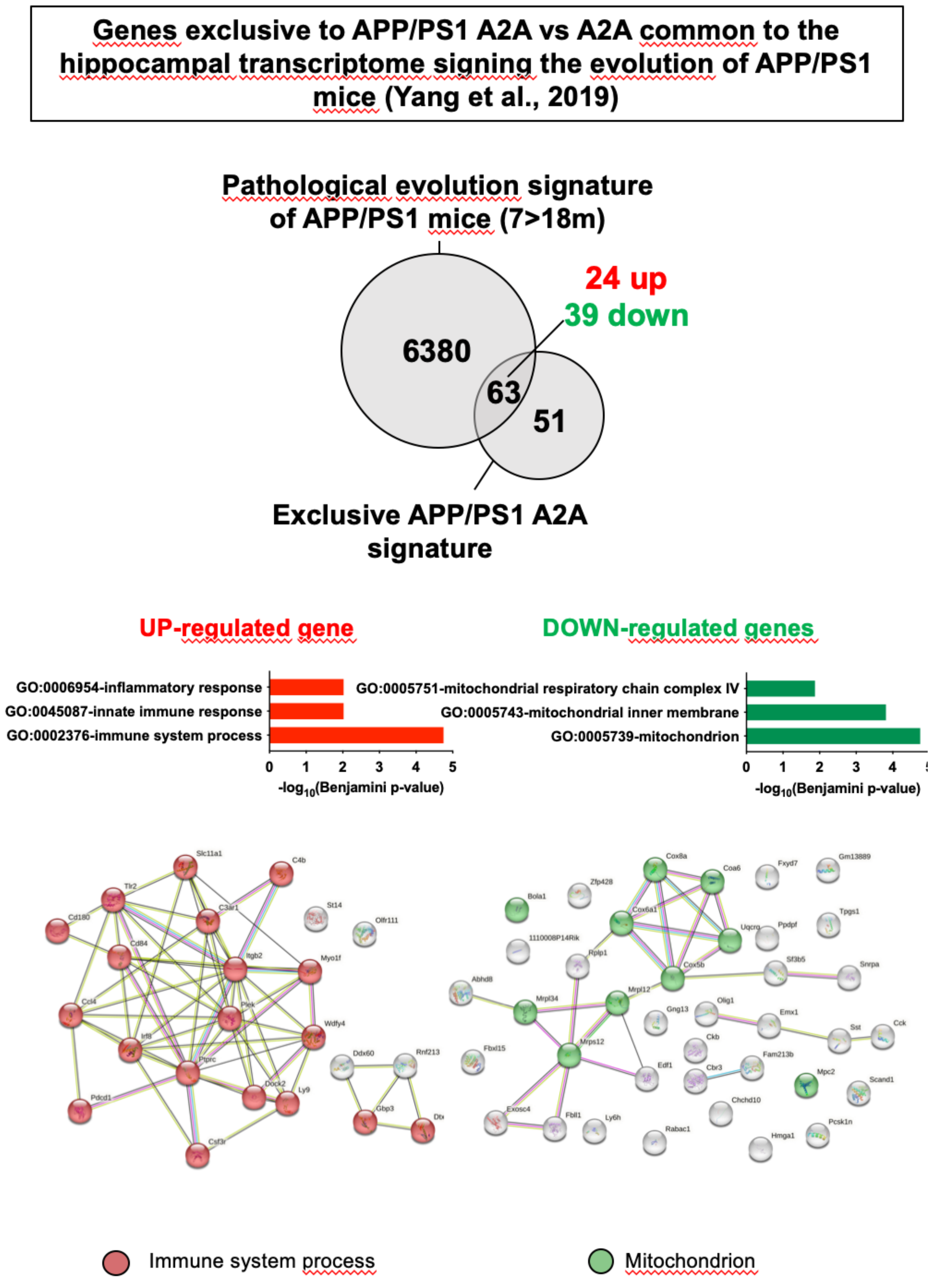

Supplementary Figure 7. Signature comparison between genes signing the evolution of hippocampal transcriptome in 18 vs 7m APP/PS1 mice and exclusive genes of APP/PS1 A2A mice using Venn diagram. Functional annotation of the 24 upregulated genes and 39

downregulated genes commonly found in evolving APP/PS1 mice and APP/PS1 A2A animals as performed with DAVID shows upregulated gene with immune-related processes (red bars) and downregulated genes with mitochondrial function (green bar). The lower panel corresponds to the STRING network analysis these genes.

Genes exclusive to APP/PS1 A2A vs A2A common to whole transcriptome hippocampal profile of late-onset AD patients (Annese et al., 2018)

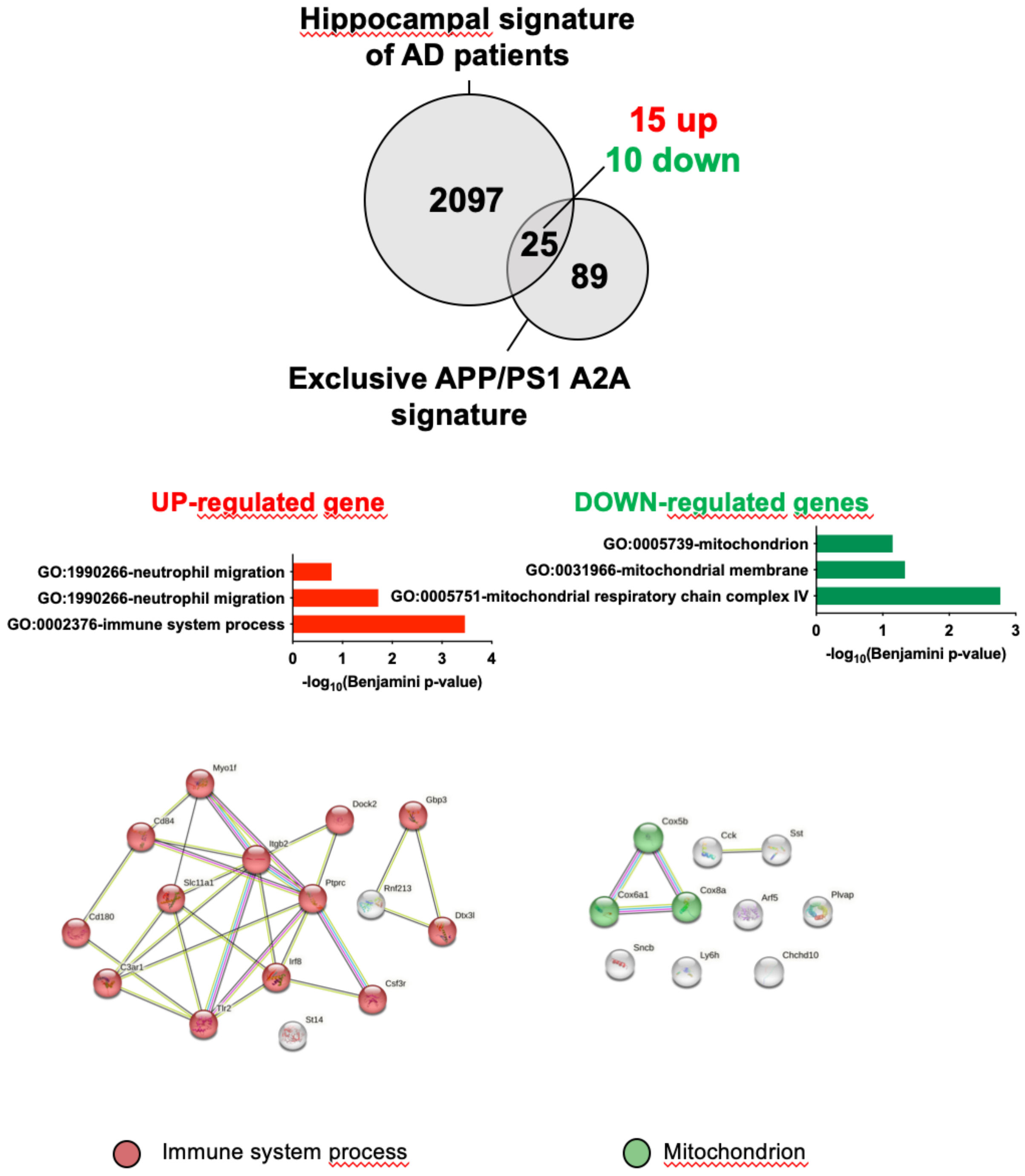

Supplementary Figure 8. Signature comparison between genes signing hippocampal gene variation of AD patients and exclusive genes of APP/PS1 A2A mice using Venn diagram.

Functional annotation of the 15 upregulated genes and 10 downregulated genes commonly found in AD patients and APP/PS1 A2A animals, as performed with DAVID, shows upregulated gene with immune-related processes (red bars) and downregulated genes with mitochondrial function (green bar). The lower panel corresponds to the STRING network analysis these genes.

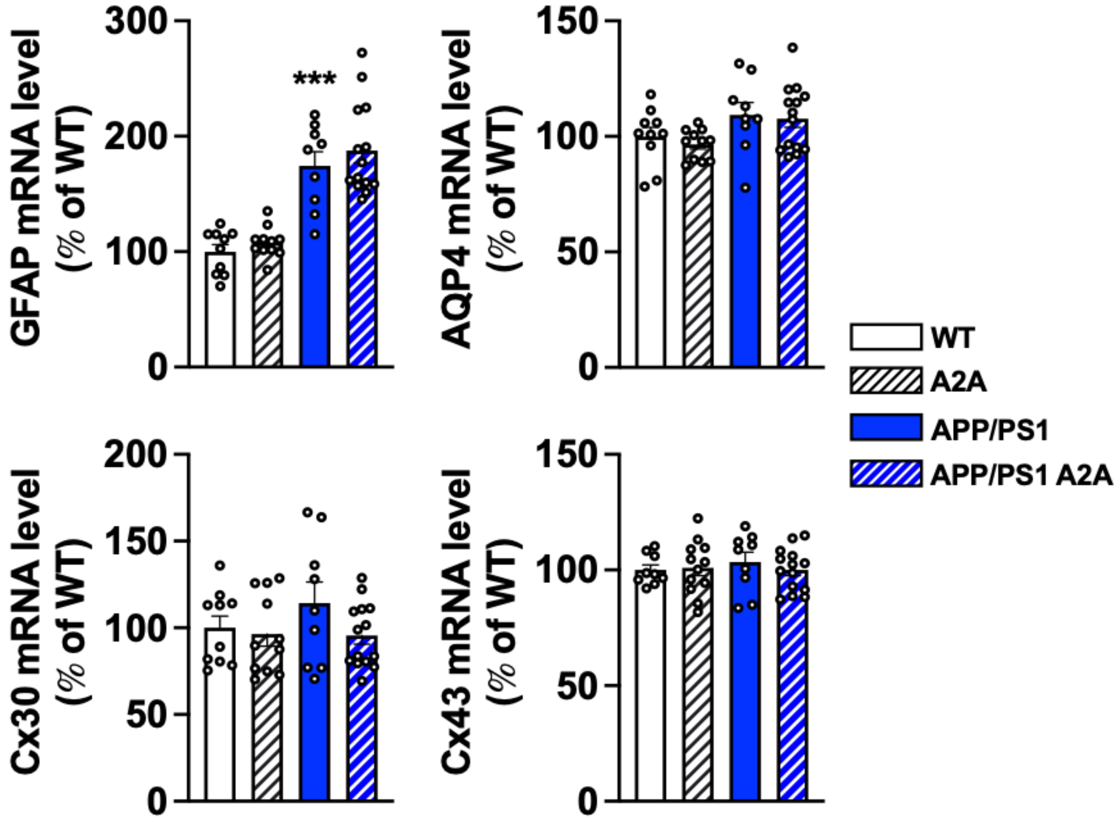

Supplementary Figure 9. mRNA levels of different astrocytic markers in the different groups of mice. *** $P<0.001$ vs. WT, One-Way ANOVA followed by Tukey's post hoc test. N=10-28 mice per group.

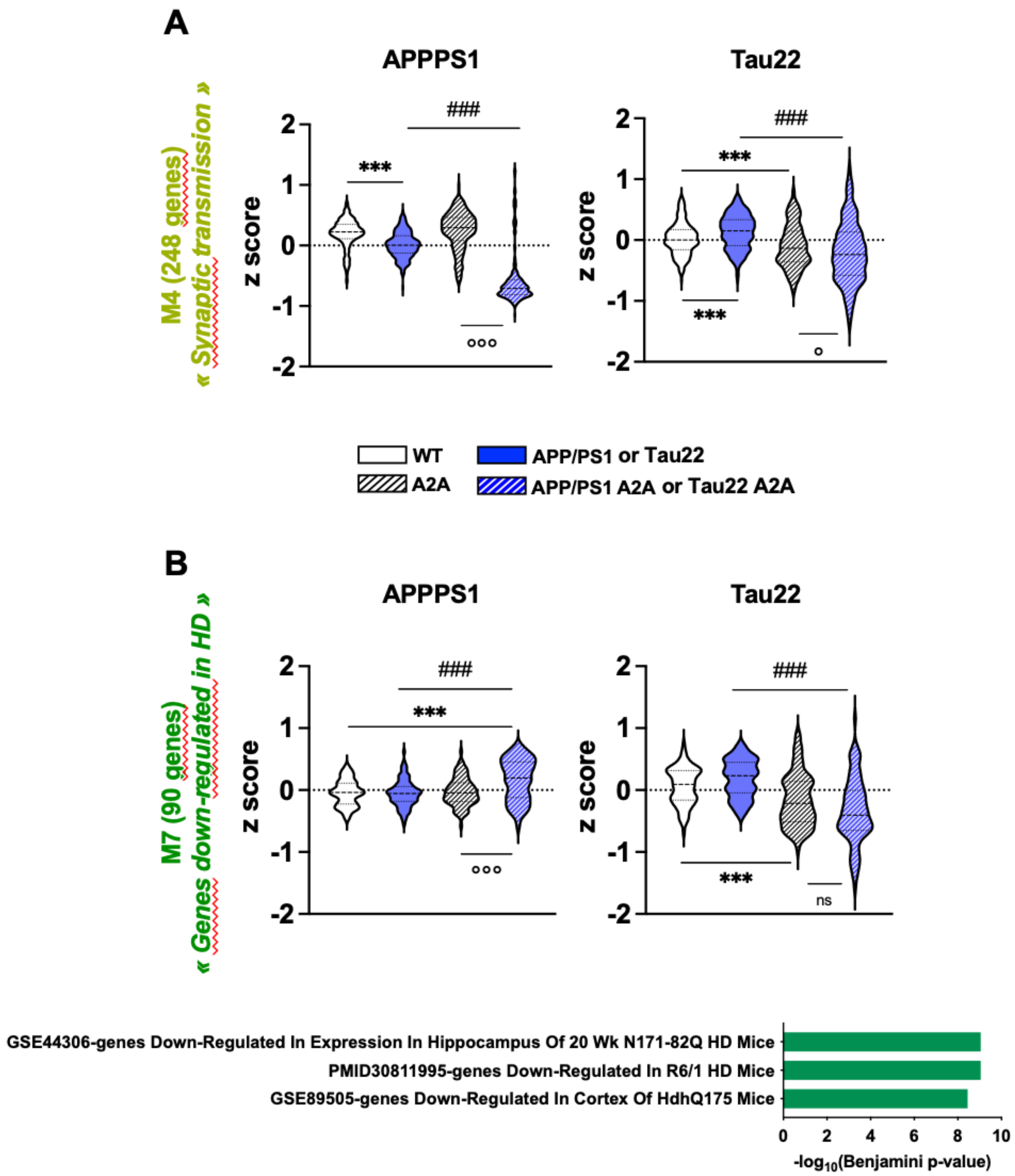

Supplementary Figure 10. Violin plots representing z-score expression values of genes from the RNA-seq analyses performed in APP/PS1 mice (this study) and THY-Tau22 mice (Carvalho et al., 2019) for the different genotypes (n = 4 per group). The M4 and M7 modules were defined using CEMITool analyses (Figure 5). **(A)** M4 module associated with “synaptic

transmission" signature. Note that gene expression in this module is down-regulated by neuronal expression of A2AR in both APP/PS1 and Tau mice, although more moderate in Tau mice. **(B)** M7 module associated with "Genes down-regulated in HD" signature. Pathway analysis (Enrichr, Biological Process) of dysregulated genes in the M7 module is represented below by the 3 most significant terms. While gene expression in this module is down-regulated in the hippocampus of several HD mouse models and down-regulated in Tau mice expressing a neuronal A2AR compared to Tau mice, it is up-regulated in the hippocampus of APP/PS1 mice expressing neuronal A2ARs compared to APP/PS1 mice. $^{***,\ \#\#\#,\ \circ\circ\circ}$ *P*< 0.001; ns: non-significant; using the non-parametric Kruskal Wallis test, original FDR method of Benjamini and Hochberg for post-hoc test.

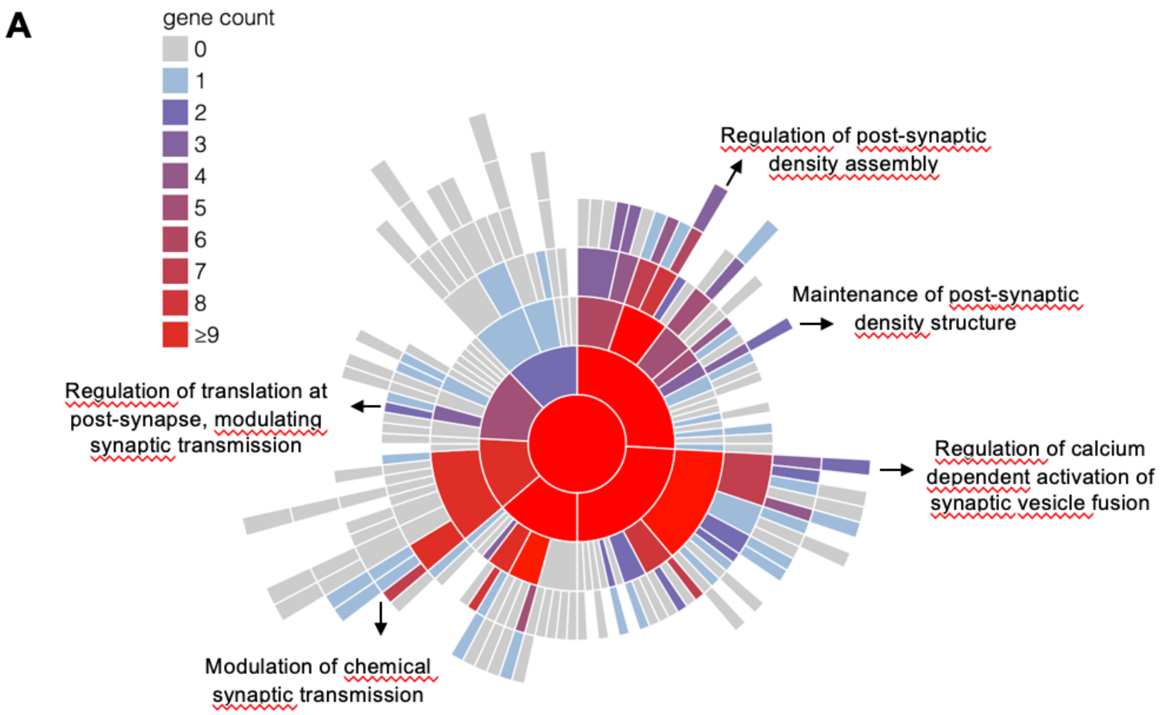

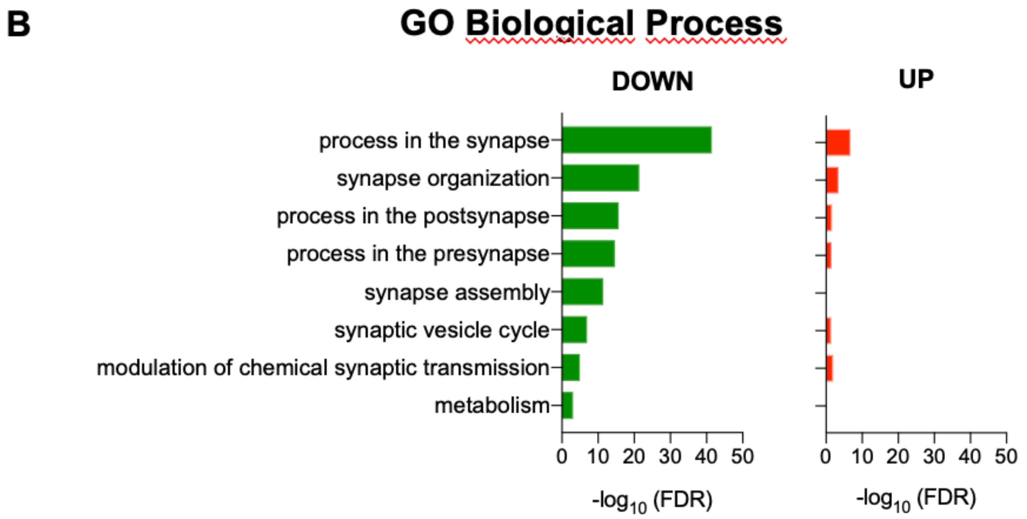

Supplementary Figure 11. SynGO analyses of proteomic results. (**A**) Significant down-regulated proteins found in APP/PS1 A2A versus APP/PS1 hippocampi. The SynGO ontologies and annotations tool revealed that 69/115 genes have a Cellular Component and 63/115 have Biological Processes annotation, among which 22 Cellular Component and 34 for Biological Processes terms are significantly enriched at 1% FDR. Warmer colors represent the predominance of proteins associated with the respective pathway. Biological Process ontology terms are specifically given for most enriched clusters. (**B**) Comparison of Biological Processes terms between up- dans down-regulated lists. Graphs showing statistics of similar Biological Process terms within the up- and down-regulated lists of the proteomic study obtained by SynGO analyses.

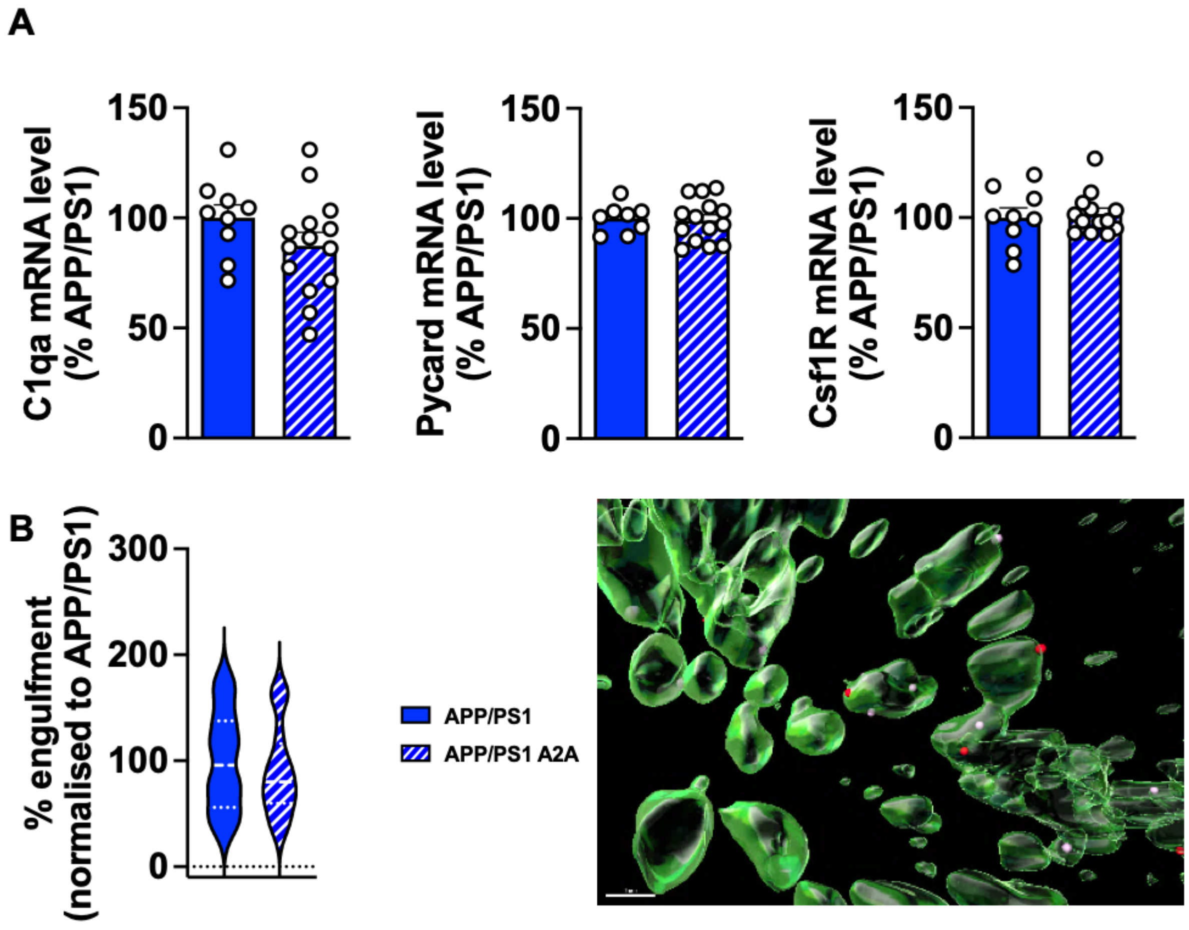

Supplementary Figure 12. Synaptic loss induced by neuronal A_{2A}R upregulation in APP/PS1 mice is not associated with a microglial-based pruning. (A) qPCR analysis of C1qa and other markers previously found upregulated in Tau A2A animals, i.e. Pycard and Csf1R showing no change in the hippocampus of APP/PS1 A2A vs. APP/PS1 mice. N=9-14 mice per group. **(B)** Quantification of engulfed Synaptophysin and Homer1 puncta within microglial Cd68^{+} microglial structures in the CA1 of APP/PS1 and APP/PS1 A2A mice. The percentage of synaptic engulfment by microglia was found unchanged. N=23-24 from 5-6 mice per group. Representative image for microglial engulfment analysis of synapses. Three dimensional reconstruction by Imaris software showing pre-synaptic (synaptophysin, in purple), post-synaptic (Homer1, in red) and lysosomal (cd68, in green) markers. (Scale bar : 5 μm).
